# Multiscale Modelling of Vascular Tumour Growth in 3D: The Roles of Domain Size and Boundary Conditions

**DOI:** 10.1371/journal.pone.0014790

**Published:** 2011-04-13

**Authors:** Holger Perfahl, Helen M. Byrne, Tingan Chen, Veronica Estrella, Tomás Alarcón, Alexei Lapin, Robert A. Gatenby, Robert J. Gillies, Mark C. Lloyd, Philip K. Maini, Matthias Reuss, Markus R. Owen

**Affiliations:** 1 Center Systems Biology, University of Stuttgart, Stuttgart, Germany; 2 Centre of Mathematical Medicine and Biology, School of Mathematical Sciences, University of Nottingham, Nottingham, United Kingdom; 3 H. Lee Moffitt Cancer Center and Research Institute, Tampa, Florida, United States of America; 4 Centre de Recerca Matemàtica, Campus de Bellaterra, Barcelona, Spain; 5 Centre for Mathematical Biology, Mathematical Institute, University of Oxford, Oxford, United Kingdom; 6 Department of Biochemistry, Oxford Centre for Integrative Systems Biology, University of Oxford, Oxford, United Kingdom; University of Arizona, United States of America

## Abstract

We investigate a three-dimensional multiscale model of vascular tumour growth, which couples blood flow, angiogenesis, vascular remodelling, nutrient/growth factor transport, movement of, and interactions between, normal and tumour cells, and nutrient-dependent cell cycle dynamics within each cell. In particular, we determine how the domain size, aspect ratio and initial vascular network influence the tumour's growth dynamics and its long-time composition. We establish whether it is possible to extrapolate simulation results obtained for small domains to larger ones, by constructing a large simulation domain from a number of identical subdomains, each subsystem initially comprising two parallel parent vessels, with associated cells and diffusible substances. We find that the subsystem is not representative of the full domain and conclude that, for this initial vessel geometry, interactions between adjacent subsystems contribute to the overall growth dynamics. We then show that extrapolation of results from a small subdomain to a larger domain can only be made if the subdomain is sufficiently large and is initialised with a sufficiently complex vascular network. Motivated by these results, we perform simulations to investigate the tumour's response to therapy and show that the probability of tumour elimination in a larger domain can be extrapolated from simulation results on a smaller domain. Finally, we demonstrate how our model may be combined with experimental data, to predict the spatio-temporal evolution of a vascular tumour.

## Introduction

Angiogenesis marks an important turning point in the growth of solid tumours. Avascular tumours rely on diffusive transport to supply them with the nutrients they need to grow and, as a result, they typically grow to a maximal size of several millimetres in diameter. Growth stops when the rate at which nutrient-starved cells in the tumour centre die balances the rate at which nutrient-rich cells on the tumour periphery proliferate. Under low oxygen, tumour cells secrete angiogenic growth factors that stimulate the surrounding vasculature to produce new capillary sprouts that migrate towards the tumour and the new vessels increase the supply of nutrients to the tissue, enabling the tumour to continue growing and to invade adjacent healthy tissue. At a later stage small clusters of tumour cells may enter the vasculature and be transported to remote locations in the body, where they may establish secondary tumours and metastases [1].

In more detail, the process of angiogenesis involves degradation of the extracellular matrix, endothelial cell migration and proliferation, capillary sprout anastomosis, vessel maturation, and adaptation of the vascular network in response to the blood flow [2]. Angiogenesis is initiated when hypoxic cells secrete tumour angiogenic factors (TAFs), such as vascular endothelial growth factor (VEGF) [3], [4]. The TAFs are transported through the tissue by diffusion where they stimulate the existing vasculature to form new sprouts. The sprouts migrate through the tissue, responding to spatial gradients in the TAFs by chemotaxis. When sprouts connect to other sprouts or to the existing vascular network via anastomosis, new vessels arise. The diameter of perfused vessels changes in response to a number of biomechanical stimuli such as wall shear stress and signalling cues such as VEGF [5], [6]. Angiogenesis persists until the tissue segment is adequately vascularised. On the other hand, vessels which do not sustain sufficient blood flow will regress and be pruned from the network [7], [8].

Tumour growth and angiogenesis can be modelled using a variety of approaches (for reviews see [9], [10]). Spatially-averaged models can be formulated as systems of ordinary differential equations (see [11], [12]). Alternatively, a multiphase approach can be used to develop a spatially-structured continuum model that describes interactions between tumour growth and angiogenesis and is formulated as a mixed system of partial differential equations (PDEs) [13]. Alternatively a 2D stochastic model that tracks the movement of individual endothelial cells to regions of high VEGF concentration is introduced in [14]. Following [14], McDougall and coworkers [15] have developed a model for angiogenesis and vascular adaptation in which the tissue composition is static and attention focusses on changes in the vasculature. This framework was then extended by Stéphanou et al. [16] to produce the first 3D simulations of angiogenesis and vascular adaptation. More recently, Macklin et al. [17] coupled a multiphase model to a discrete model of angiogenesis that accounts for blood flow, non-Newtonian effects and vascular remodelling. The models are coupled in two ways: via hydrostatic pressure which is generated by the growing tumour and acts on the vessels, and via oxygen which is supplied by the vessels and stimulates growth. Lloyd et al. [18] have developed a model for neoplastic tissue growth which accounts for blood and oxygen transport, and angiogenic sprouting. The strain (local deformation) in the tumour tissue is assumed to be an increasing function of the local oxygen concentration. In separate work, Owen et al. [19], building on the work of Alarcón and co-workers [20]–[23], proposed a 2D multiscale model for vascular tumour growth which combines blood flow, angiogenesis, vascular remodelling and tissue scale dynamics of multiple cell populations as well as the subcellular dynamics (including the cell cycle) of individual cells.

While several two-dimensional models of angiogenesis account for tumour growth, few groups consider vascular tumour growth in three space-dimensions. In an extension to work by Zheng at al. [24], Frieboes et al. [25] couple a mixture model to a lattice-free continuous-discrete model of angiogenesis [26] to study vascular tumour growth. However, the effects of blood flow and vascular remodelling are neglected. Lee et al. [27] studied tumour growth and angiogenesis, restricting vessel sprouting to the tumour periphery and surrounding healthy tissue. They incorporated vessel dilation and collapse in the tumour centre, and analysed the micro-vessel density within the tumour. Building on work by Schaller and Meyer-Hermann [28], Drasdo et al. [29] developed a lattice-free model for 3D tumour growth and angiogenesis that includes biomechanically-induced contact inhibition and nutrient limitation. However, they do not consider an explicit cell cycle model, they neglect the effects of flow-induced vascular remodelling and ignore interactions between normal and tumour cells. Similarly Shirinifard et al. [30] present a 3D cellular Potts model of tumour growth and angiogenesis in which blood flow and vascular remodelling are neglected, as are the cell cycle and competition between normal and tumour cells.

In this paper, we extend the multiscale model proposed by Owen et al. [19] from 2D to 3D. In contrast to former models we relax the assumption that adaptation of vessel diameters occurs on a timescale which is much shorter than the tumour doubling time and assume instead that adaptation occurs on the same timescale as tumour cell movement and proliferation. We perform extensive numerical simulations of our model to investigate how the 3D structure influences the growth and composition of the tissue and its vascular network. We start by considering a 2D domain in which the tissue (either normal cells only, or normal cells with a small tumour implanted) is initially perfused by two parallel vessels, and then increase the extent of the domain in the third dimension. We then focus on whether it is possible to extrapolate simulation results obtained for small subsystems to larger ones, and the extent to which these results depend on the choice of boundary conditions. This is important because computational limitations often mean that it is not feasible to simulate large spatial domains. Hence we implement periodic boundary conditions in either the 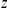-direction or in both the 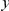
- and 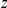-directions (as far as we are aware such boundary conditions have not been implemented in previous individual-based models for angiogenesis). We perform further simulations to investigate the tumour's response to therapy and present a strategy to extrapolate tumour elimination probabilities to larger domains. We also show how vascular networks derived from experimental data can be integrated into our model.

## Methods

**Figure 1:**
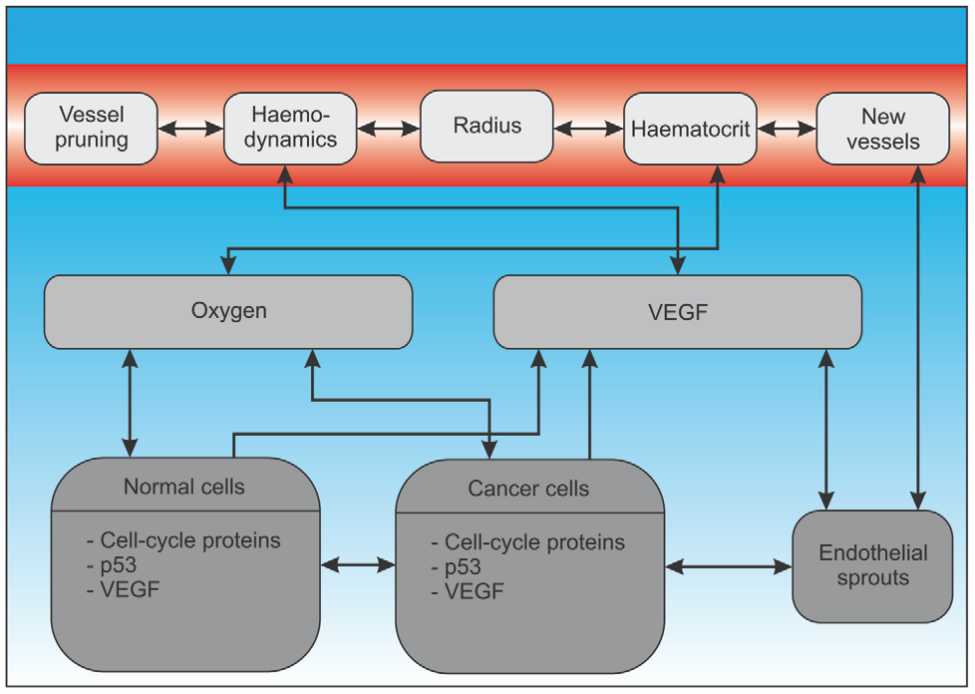
Multiscale model overview (interaction diagram). This figure shows the connections between the different modelling layers. In the subcellular layer the cell cycle protein concentrations and the p53 and VEGF concentrations are modelled via systems of coupled ordinary differential equations. The local external oxygen concentration influences the duration of the cell cycles. Cells consume oxygen, and produce VEGF in the case of hypoxia. Extracellular VEGF also influences the emergence of endothelial sprouts and their biased random walk towards hypoxic regions. If endothelial sprouts connect to other sprouts or the existing vascular network, new vessels form. Vessel diameter is influenced by the local oxygen concentration and flow-related parameters, such as pressure and wall shear stress. The vascular network delivers oxygen throughout the tissue.

The computational model describes the spatio-temporal dynamics of tumour growth in a vascular host tissue. Cells are represented as individual entities (agent-based approach) each with their own cell cycle and subcellular-signalling machinery. Nutrients are supplied by a dynamic vascular network, which is subject to remodelling and angiogenesis. The interactions between the different modules are depicted in Figure 1.

**Figure 2:**
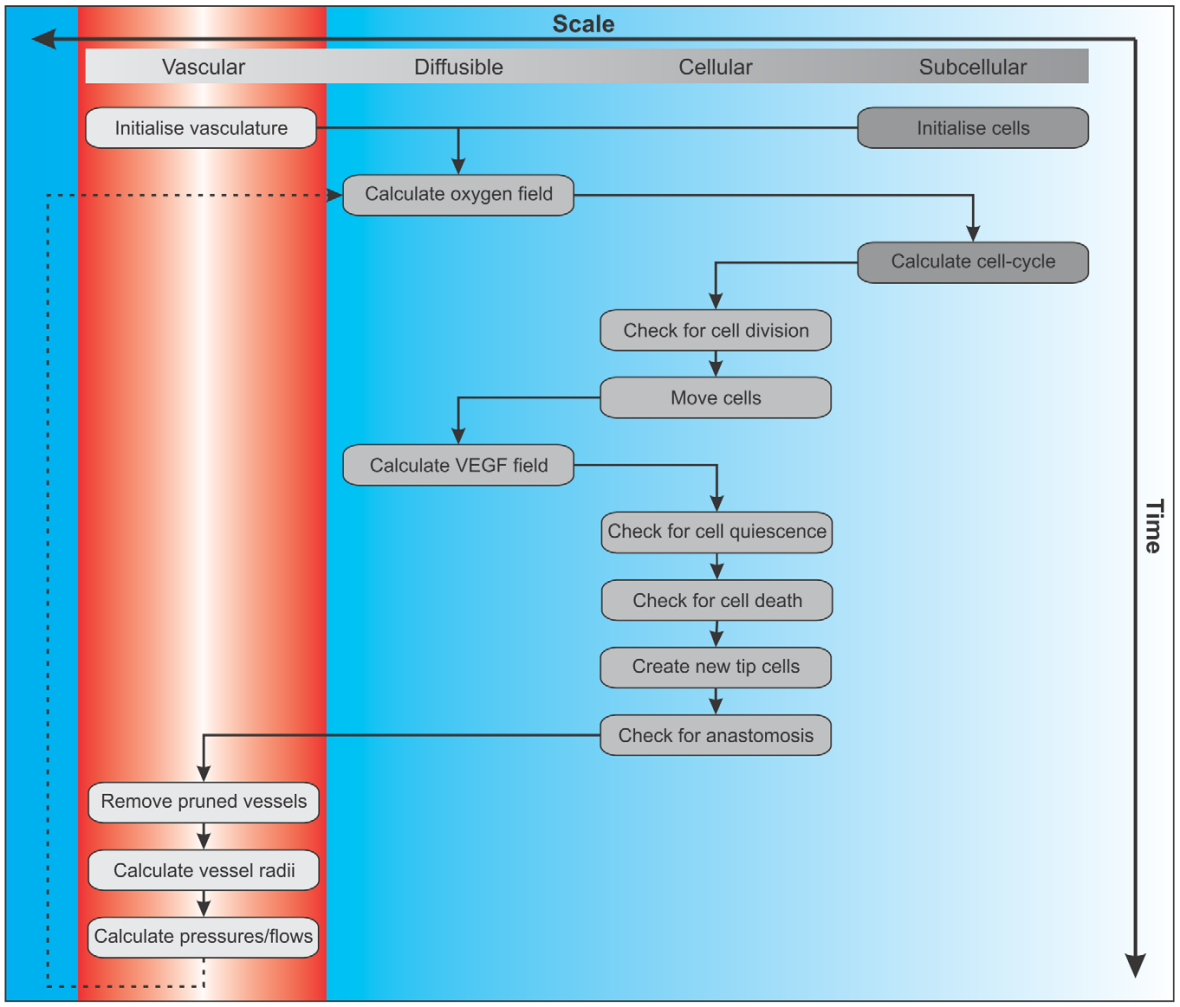
Multiscale model overview (flowchart). The flowchart shows the temporal sequence of the computational steps in our simulation.

Our model is formulated on a regular grid that subdivides the simulation domain into lattice sites. Each lattice site can be occupied by several biological cells whose movement on the lattice is governed by reinforced random walks, and whose proliferation is controlled by a subcellular cell cycle model. The vascular network consists of vessel segments connecting adjacent nodes on the lattice, with defined inflow and outflow nodes with prescribed pressures. We also specify the amount of haematocrit entering the system through the inlets. The vessel network evolves via (i) sprouting of tip cells with a probability that increases with the local VEGF concentration, (ii) tip cell movement is described by a reinforced random walk, and (iii) new connections forming via anastomosis. In addition, vessel segments with low wall shear stress may be pruned away. Elliptic reaction-diffusion equations for the distributions of oxygen and VEGF are implemented on the same spatial lattice using finite difference approximations, and include source and sink terms based on the location of vessels (which act as sources of oxygen and sinks of VEGF) and the different cell types (e.g. cells act as sinks for oxygen and hypoxic cells as sources of VEGF). The flowchart in Figure 2 summarises the algorithm and shows how processes that act on different space and time scales are accommodated. In summary, after initialising the system, the diffusible fields, cellular and subcellular states are updated (including cell division and movement), before the vessel network is updated, this process being repeated until the simulation ends. Further details about each step of the computation and the model parameter values can be found in the Text S1. The model is implemented in C++, using CVODE (https://computation.llnl.gov/casc/sundials/main.html) to integrate the subcellular ODEs, and SuperLU (http://crd.lbl.gov/~xiaoye/SuperLU/) to solve the linear systems for the flow calculation.

It was necessary to implement several changes to the computational algorithm when extending the model from 2D to 3D. For example, in order to reduce the calculation time of the diffusion fields, the resulting linear system of equations is now solved with an iterative GMRES-solver, rather than the direct SuperLU-solver which was used in [19] for 2D simulations. We remark that the linear system of equations for the flow calculation is still solved with the SuperLU solver as higher accuracy of solutions is needed to determine whether vessels are unperfused or if there is low flow. Adaptation of vessel radii can be observed on two different timescales. The first represents an acute response (short timescale, with a magnitude of minutes or hours) to external or internal stimuli (i.e. vessel constriction). The second timescale acts on a longer timescale (days) and includes vessel maturation. Based on this consideration, our vessel-adaptation algorithm is altered so that vessel radii are no longer iterated to steady-state at each time-step (30 min). Instead, they evolve on each time-step, reflecting the assumption that vessel adaptation occurs on the same timescale as cell movement and proliferation. Periodic boundary conditions are implemented for the random walks of the cells (including endothelial tip cells), for the vessel network and flow calculation, and for the reaction-diffusion equations. In order to integrate more closely with experiments, an interface is implemented to import experimentally-derived vascular network data, in order to specify the initial vasculature and then apply the tumour growth and angiogenesis model. The 3D results are visualised in Povray (http://www.povray.org) and OpenInventor (http://oss.sgi.com/projects/inventor/).

Stochastic components of the model include endothelial tip cell emergence and random walks of different cell types, with trajectories generated from a sequence of random numbers (we use the Mersenne Twister algorithm, http://www-personal.umich.edu/~wagnerr/MersenneTwister.html). In order to assess the degree of stochasticity in our model simulations, we carried out multiple simulations using different seeds for the random number generator. In the next section we will study the variation in mean values of our stochastic simulations as, for example, domain size varies. Therefore it is necessary to check if these mean values really differ significantly or if they only represent different samples that belong to the same probability density. We estimate the confidence intervals of our mean values by applying a bootstrapping method—lternatively one could apply non-parametrical methods, such as the Wilcoxon-Mann-Whitney-Test [31]. The bootstrapping method enables us to plot 95% confidence bands for the mean dynamics in time and confidence intervals of the mean long-time values.

## Results

### 3D vascular tumour growth

**Figure 3:**
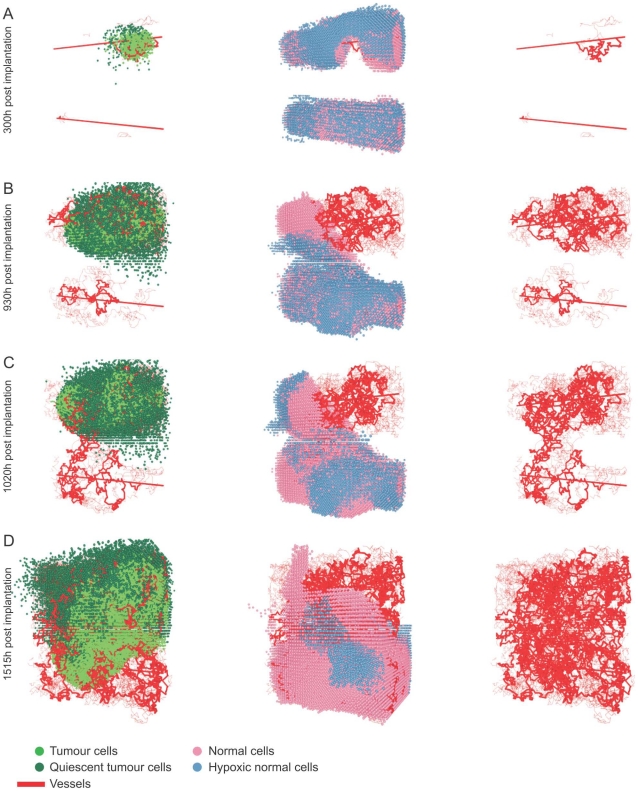
Tumour growth in healthy tissue. The tumour cells and vasculature are depicted in the left column (see Video S1), the vasculature and normal cells in the middle column (Video S2) and the vessel network in the right column. The figure shows a realisation of a 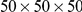 domain with a cube of tumour cells implanted in healthy tissue with two straight initial vessels. Most tumour cells become quiescent and then die. Thereafter the following steps occur: **A**) Vessels emerge near the initial tumour and form a well-vascularised tumour; far from the two initial vessels most normal cells have died, leaving two cylindrical shaped cell populations around the vessels. **B**) The tumour grows along the direction of maximum oxygen supply and displaces the normal cells. **C**) An important step in the tumour development – the first bridge between the upper and lower network is built. **D**) Once a connection has been made between the upper and lower vessels, the tumour is able to colonise the lower part of the simulation domain.

The results from a typical simulation showing the development of a tumour and its associated network of blood vessels are depicted in Figure 3. Simulations were performed on a 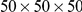 lattice with spacing 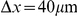, which corresponds to a 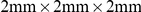 cube of tissue. For the following simulations, each lattice site can be occupied by at most one cell (either normal or cancerous), which implies that, for the grid size used (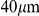), the tissue is loosely packed. A small tumour was implanted at 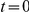 in a population of normal cells perfused by two parallel parent vessels with countercurrent flow (i.e. the pressure drops and hence flows are in opposite directions). Initially, insufficient nutrient supply in unvascularised areas causes widespread death of the normal cells. The surviving tumour cells reduce the p53 threshold for death of normal cells (see Equation (8) in Text S1), which further increases the death rate of the normal cells and enables the tumour to spread. Initially, most of the tumour cells are quiescent and secrete VEGF which stimulates an angiogenic response. After a certain period of time the quiescent cells die and only a small vascularised tumour remains encircling the upper vessel. The tumour expands preferentially along this vessel, in the direction of highest nutrient supply. Diffusion of VEGF throughout the domain stimulates the formation of new capillary sprouts from the lower parent vessel. When the sprouts anastomose with other sprouts or existing vessels, the oxygen supply increases, enabling the normal cell population to recover. Because the tumour cells consume more oxygen than normal cells, and they more readily secrete VEGF under hypoxia, VEGF levels are higher inside the tumour and the vascular density there is much higher than in the healthy tissue. The tumour remains localised around the upper vessel until new vessels connect the upper and lower vascular networks. Thereafter the tumour cells can spread to the lower region of the domain until eventually the domain is wholly occupied by cancer cells and their associated vasculature.

We remark that several of the parameter values used to produce the simulation results presented in Figure 3 differ from those used previously to generate 2D simulations [19]. For example, we increased the rate of oxygen transport 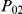 from the vasculature into the tissue. Without this change, all tumour cells died after a short period of time. With the parameters from the two-dimensional model [19] almost no new vessels are formed, as the time for vessels to meet before they die (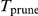) is too short given the longer paths that endothelial tip cells can take in three space dimensions. We also decreased 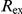, the size of the radius surrounding a vessel sprout in which new sprouts are not permitted to emerge (see Table 5 in Text S1). Without this change only a few vessels form around each parent vessel, and the tumour cells are unable to colonise the whole domain (after 4000 h of simulated time, the cells remain localised around each parent vessel).

**Figure 4:**
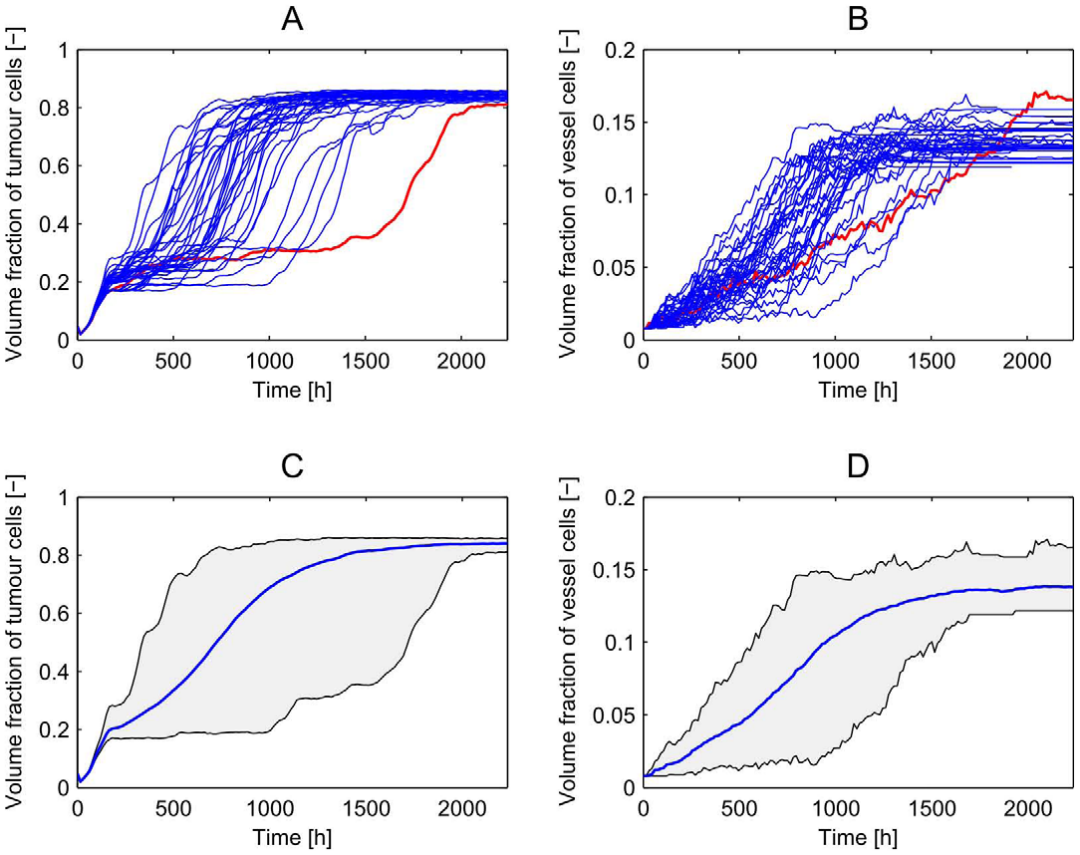
Multiple realisations of angiogenesis simulations. **A,B**) the time courses of tumour and vessel volume fractions for 25 realisations of our multiscale model of vascular tumour growth performed in a 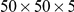 domain. One can clearly see the highly stochastic nature of the process. Sometimes there is a long lag-phase before the tumour starts to grow exponentially (see red line in A) and B)). **C,D**) depict how the mean behaviour is located in the domain that is spanned by all simulations.

Figure 4 shows the time course of the tumour and vessel volume fractions for these 25 realisations. The volume fractions are defined by the ratio of lattice sites occupied by tumour or vessel cells to the total number of lattice sites. The high degree of variability in these results explains why it is necessary to average over several realisations in order to draw robust conclusions from our simulations.

### Dependence on the 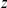-extent

**Figure 5:**
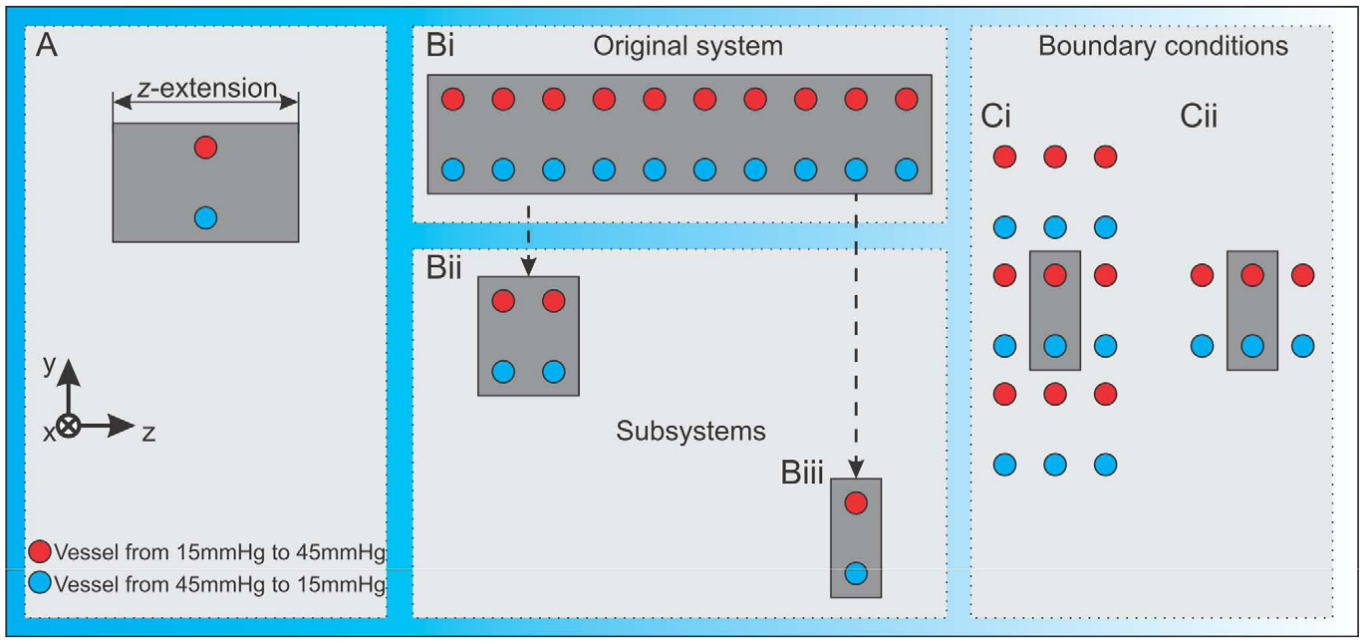
Schemes of numerical studies. **A**) We consider two parallel vessels running in the 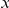-direction and centred with respect to the variable 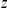-extension, with equal mean pressures and opposite pressure drops. **B**) We analyse how the interference between neighbouring subsystems influences the behaviour in a larger domain: **Bi**) We consider a large (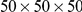) domain, where all 10 subdomains, each with two parent vessels, are allowed to interfere with each other and farfield communication is incorporated in this setting. **Bii**) Depicts a 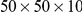 subsystem with two parent vessel pairs. Thus nearfield (but not farfield) interaction in the 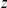-direction is now included. **Biii**) The most restricted example, a single 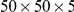 subdomain, thus preventing all communication in the 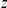-direction. In A) and B) we apply reflecting (zero-flux) boundary conditions. **C**) Different boundary conditions are considered: **Ci**) Periodic boundary conditions in the 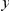
- and 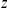-direction. The grey subdomain is effectively surrounded by other networks. **Cii**) Periodic boundary conditions in the 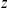-direction only, which should produce similar behaviour to Bi).

**Figure 6:**
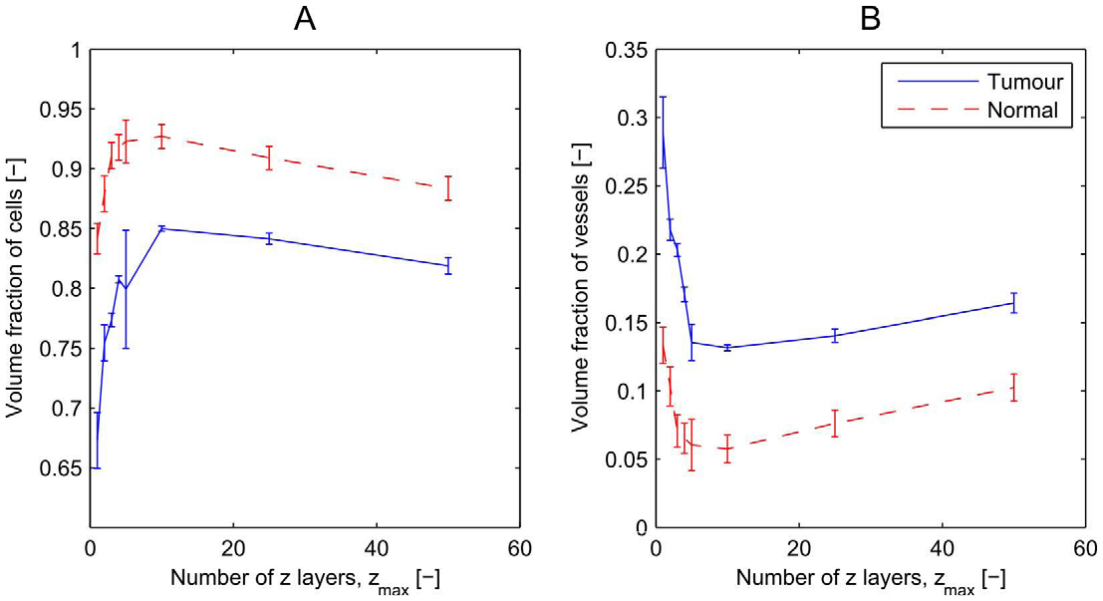
Enhancement of the 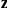
 **-extent.** For the numerical experiments indicated in Figure 5(A), on a 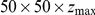 domain, we show: **A**) the volume fraction of normal cells (red line, for simulations with normal cells only) or cancer cells (blue line, for simulations where a tumour is implanted) at their long-time value averaged over several simulations. Both cell densities increase as 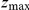 increases from 1, until a maximum is reached at 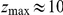. For larger values of 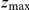 the cell density decreases again. **B**) the associated vascular volume fractions.

In order to determine the effect of extending the model from 2D to 3D we performed simulations in which the domain size is varied in the 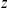-direction (see Figure 5(A)). Thus, we considered computational grids of size 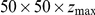, and placed two parent vessels at the same 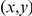 co-ordinates and centred in the 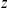-direction (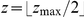). We ran simulations with normal cells only (as a control), or with a small tumour implanted into a population of normal cells. In the latter case the normal cells are always eliminated and the tumour invades the entire domain. Figure 6 shows the cell and vessel volume fractions averaged over several realisations for each domain size considered at time 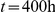. Increasing 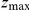 leads to an increase in the normal (or tumour) cell volume fraction, with a corresponding decrease in the vascular volume fraction. The normal (or tumour) cell volume fraction reaches a maximum when 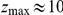 (when the domain size in the 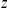-direction is about 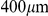) and then decreases for larger values of 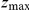. The vascular volume fraction exhibits a complementary dependence on 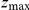, decreasing as 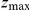 increases from 1, before reaching a minimum when 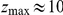 and increasing for larger values of 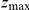. This behaviour can be explained as follows.

The 2D network for 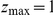 is capable of nourishing 3D domains with several cell layers in the 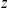-direction, because the maximum distance of cells from the plane of the parent vessels is less than the characteristic length scale associated with the combined effects of oxygen diffusion and consumption. Thus the 2D network (with 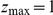) is capable of nourishing more cells with the same number of vessels. Consequently the ratio of normal (or tumour) cells to vessels increases rapidly as 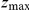 increases from 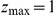. For larger values of 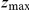, the vascular network must expand in the 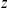-direction to provide the normal (or tumour) cells with the nutrients they need to remain viable and colonise the entire domain. This behaviour can be seen by considering the total vascular volume (rather than density) as a function of 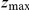: for small 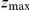 the vascular volume increases only weakly with 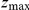, but for 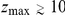 the vascular volume increases rapidly with 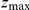 (results not shown).

As noted earlier, in our simulations the vessel density in the tumour is higher than in normal tissue because the tumour cells consume more oxygen, and, as a result, require a higher vascular density to meet their nutritional requirements. In addition, the tumour cells start to produce VEGF at higher oxygen concentrations than normal cells (i.e. they require a lesser degree of hypoxia to stimulate VEGF release), so that overall VEGF production is significantly higher in the tumour. This elicits a stronger angiogenic response in the tumour than in the normal tissue because the sprouting probability increases with the VEGF concentration (Equation (11) in Text S1).

### Isolated and coupled subsystems and the role of boundary conditions

Here we focus on establishing whether it is possible to extrapolate simulation results obtained for small subsystems to their associated larger ones. An overview of the numerical experiments that we perform is given in Figure 5(B). We choose three different domains for our numerical study, each possessing a certain degree of symmetry, and each with reflecting (zero-flux) boundary conditions. In the first case (Figure 5(Bi)), we consider a 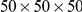 domain, which initially contains 10 pairs of vessels that are parallel to the 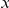-axis and equally spaced in the 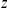-direction. The cells, diffusible substances and new angiogenic vessels in the neighbourhood of each vessel pair permit nearfield and farfield interactions throughout the domain. In particular, new vessels may connect adjacent vessel pairs (nearfield interaction), or the whole vascular network can be connected across the full extent of the domain (farfield interaction). In the second case (Figure 5(Bii)), we consider a 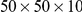 domain which is initially perfused by only two vessel pairs. Nearfield interactions between neighbouring subsystems can occur, but long range interactions cannot. Finally, we consider a 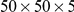 domain which initially comprises only one vessel pair, so that vessel-vessel interactions in the 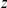-direction can not occur (Figure 5(Biii)).

**Figure 7:**
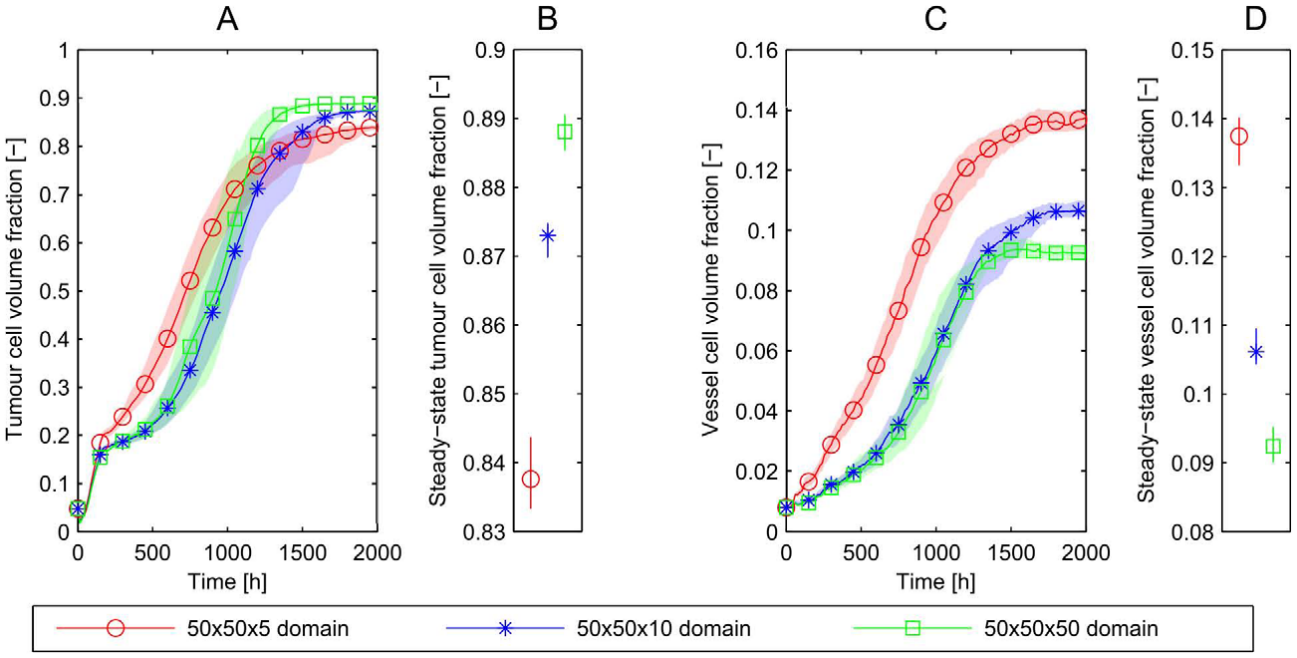
Growth kinetics in isolated and coupled subsystems. For the numerical experiments indicated in Figure 5(Bi)–(Biii), we show: **A**) The tumour cell volume fraction over time. After the first death of some tumour cells the tumour recovers and the increasing amount of oxygen supported by the vascular system promotes further growth. After the first phase of rapid growth, the tumour cells can grow fastest in the smallest domain. We obtain a different final tumour cell fraction (it is lowest on the smallest domain). **C**) The vessel volume fraction. Note that the largest domain has the most efficient oxygen supply to the tissue and thus the highest tumour cell to vessel ratio. We have also plotted the 95% confidence bands for the mean dynamics in time (A,C) by applying a bootstrapping method [31]. From these confidence intervals we conclude that the differences between the mean values in the long-time behaviour are statistically significant. **B**) **and D**) show the confidence intervals of the long-time values of the tumour and vessel cell volume fractions.

In Figure 7, we compare the behaviour of the three cases introduced above, results being averaged over 25 simulations. If there is no interaction in the 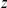-direction then all three cases should yield similar results, and the single subsystem would provide a good representation of the larger domain. However, if the averages differ then we conclude that larger 3D domains are needed to obtain results that incorporate all spatial effects and accurately represent the behaviour of the system over a longer timescale.

Figure 7 shows that tumour growth and vessel network formation are fastest in the smallest domain and the vessel density at long times is higher than for the two larger domains. In the 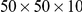 domain, with initially two parallel vessel networks in the 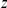-direction, growth is slower and the final vessel density lower than in the largest domain, with 10 vessel-pairs in the 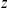-direction, vessel growth is slowest and the final vessel network is least dense. We remark that the simulations on all three domains have equivalent initial conditions, and are similar at early times, until the first new blood vessels are created. The oxygen field then becomes asymmetric, causing local changes in cell cycle duration and rates of VEGF secretion. These changes influence the subsequent dynamics, causing the system to become more asymmetric. Thereafter we can no longer compare solutions in the smaller subsystems with those for the full domain. We note also that differences between the results for the 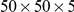 domain (no interaction) and the 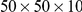 domain (nearfield interaction only) are much greater than those between the 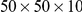 domain and the 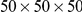 domain (nearfield and farfield interaction). Hence, for the parameter values chosen here, nearfield interactions have a stronger influence on the simulation results than farfield interactions.

It was not possible to reproduce the behaviour of the large domain by the small domain. In order to determine whether this result is due to our choice of boundary conditions we implemented periodic boundary conditions. Periodic boundary conditions allow vessel connections to “wrap around” the domain and mimic connections between subsystems in larger domains. As before simulations are carried out in three different settings. In the first case (where reflecting/zero-flux boundary conditions are imposed as discussed above), angiogenic sprouts are reflected at boundaries (endothelial tip cells and other cell types must move along the domain boundary or back into the domain). In the second case, singly-periodic boundary conditions are applied: the domain is periodic in the 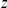-direction and reflecting in the 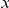
- and 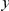-directions (see Figure 5(Cii)). In the third case, doubly periodic boundary conditions are periodic in the 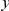
- and 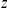-directions, and reflecting in the 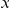-direction (see Figure 5(Ci)).

**Figure 8:**
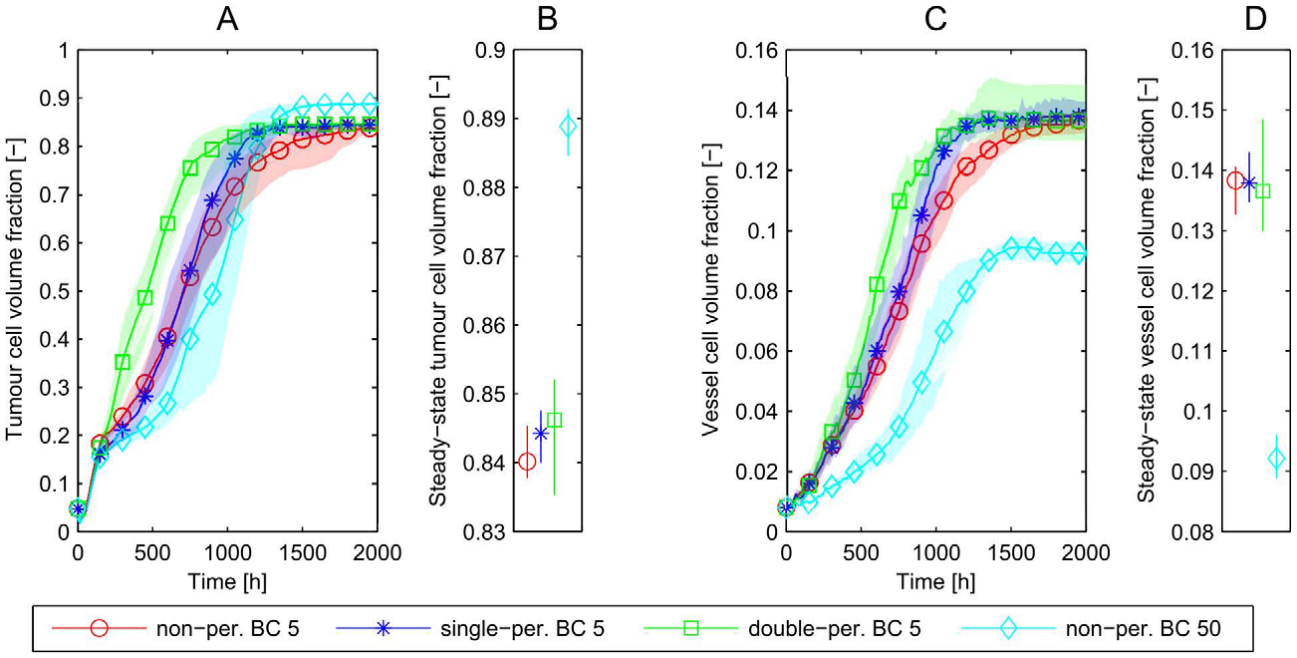
Simulations with different boundary conditions. To analyse the discrepancy (illustrated in Figure 7) between the results in the isolated small (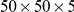) subdomain and the large (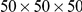) simulation domain in more detail, different boundary conditions are implemented and their influence is studied. In particular, we compare a subset of the numerical experiments indicated in Figure 5: (Bi) non-periodic BC, 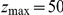; (Biii) non-periodic BC, 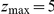; (Ci) double-periodic BC, 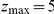; (Cii) single-periodic BC, 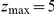. For all three types of boundary condition, the simulations lead to similar final vessel densities for the small domains (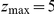), which still differ significantly from the large domain. Hence, for this initial subdomain vasculature and with the various possible choices of boundary conditions, the smallest domain cannot be representative of the larger domain.

In Figure 8 we compare results for the large (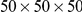) domain with those for the smallest domain (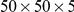) for the three different choices of boundary conditions. We have already seen that the small domain with non-periodic (reflecting) boundary conditions does not provide a good representation of the behaviour of the larger domain. Figure 8 shows that neither singly-periodic nor doubly-periodic boundary conditions alter this outcome, in all three cases the final vessel density for the small domain does not approach the density of the large domain. In conclusion, for this choice of initial vascular network (pairs of parallel vessels with countercurrent flow), and for the boundary conditions considered, simulations on the smallest domain are not representative of the system dynamics on larger domains. Before discussing why this occurs we consider whether the discrepancies persist for more complex choices of the initial vascular network.

### Multi-vessel basic tissue unit

**Figure 9:**
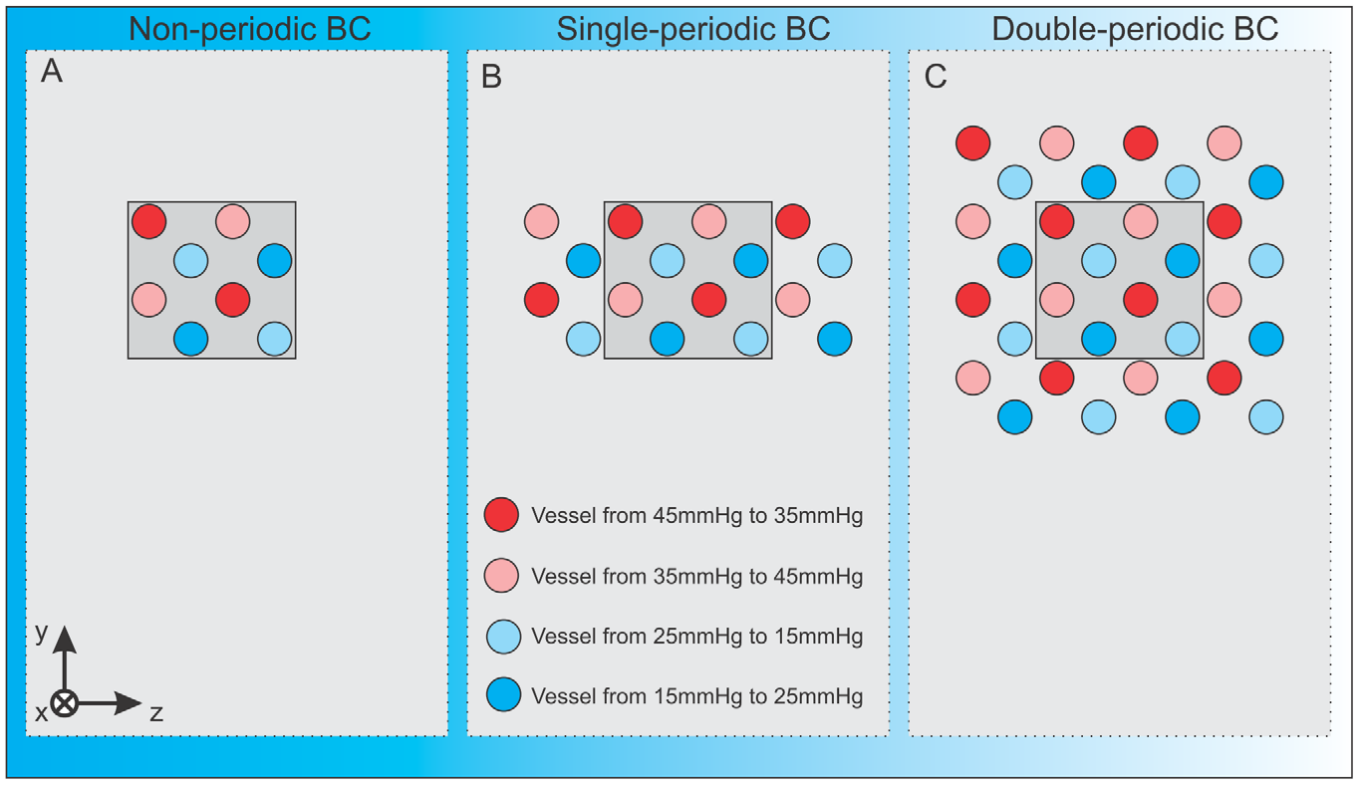
Multi-vessel basic vascular unit. **A**) A basic parent vessel unit that includes eight vessels with equal pressure differences, but with four different mean pressures, arranged so that the nearest parent vessel in each case is at a different mean pressure, which should enable functional vessels to form within and between subdomains. Non-periodic means that we consider reflection boundary conditions in all directions. **B**) Periodic boundary conditions in the 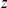-direction. **C**) Periodic boundary conditions in the 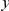
- and 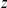-directions.

Guided by the results presented above, we now consider a subsystem that is initially characterised by multiple vessels with different pressures and flow orientations. Figure 9(A)–(C) shows the initial distribution of vessels and the different boundary conditions. The basic subsystem now comprises eight parent vessels with equal pressure differences along their lengths, but with four different mean pressures, arranged so adjacent vessels are at different mean pressures. This configuration is designed to enable functional vessels to form within and between subdomains. This contrasts with the previous case where adjacent vessels were at equal mean pressures, which promoted pruning of connections between subdomains.

**Figure 10:**
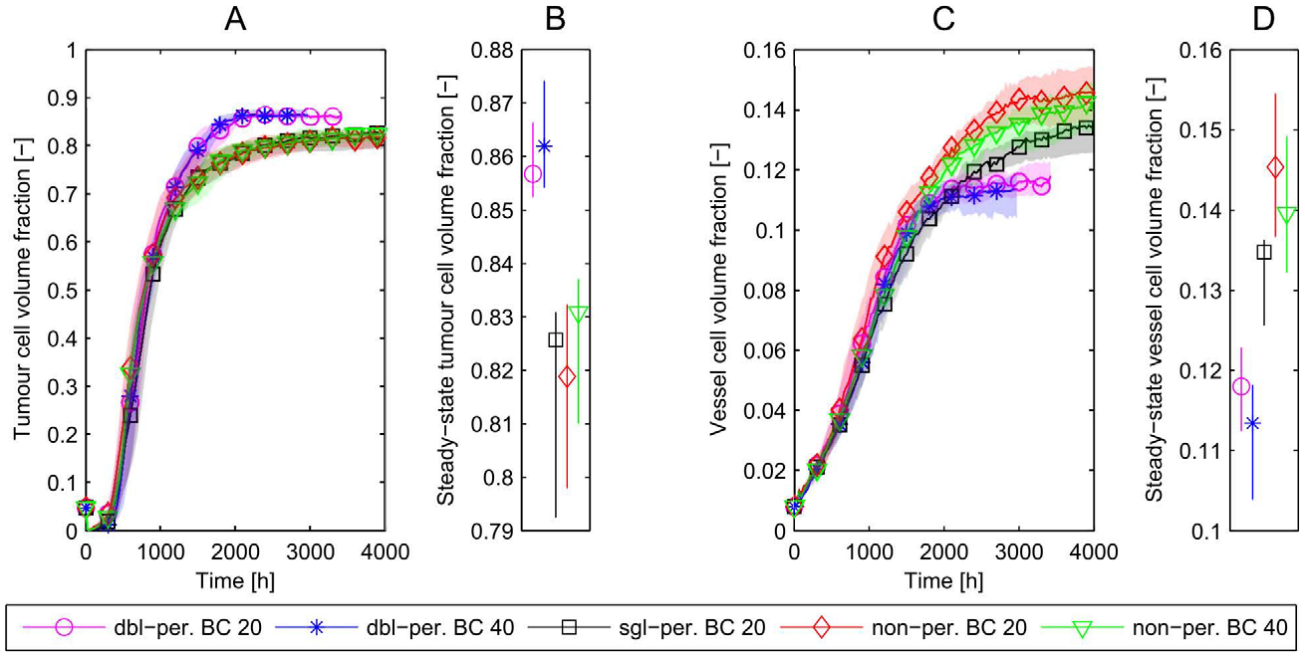
Multi-vessel basic vascular unit simulations. **A–C**) Simulations in a 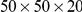 domain are compared to a 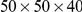 domain for each combination of boundary conditions. We find that the domain size has only a weak effect, but the choice of boundary conditions makes a significant difference to the long term tumour cell and vessel volume fractions. The most efficient vascular network can be formed on the doubly-periodic domain, while we get the least efficient network for the non-periodic boundary conditions. **B**) and **D**) show the long-time values with 95% confidence intervals.

In order to determine whether the multi-vessel subdomains are representative of larger domains, simulations with equivalent initial distributions of vessels but different domain sizes are performed. Averaged results from 10 simulations are presented in Figure 10(A)–(B). Regardless of the choice of boundary conditions, simulations on a 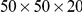 domain yield results which are equivalent to those for a 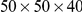 domain.

Further, in this case, the choice of boundary conditions (rather than the number of, or interactions between, subsystems) has a strong effect on the growth and composition of the tumour and the associated long-time volume fractions. Figures 10(A) and (B) show that doubly-periodic boundary conditions generate the most efficient networks in terms of greatest ratio of tumour cell volume to vascular volume, whereas non-periodic boundary conditions lead to the least efficient. We explain these results in the following way: doubly-periodic boundary conditions give rise to an initial vessel distribution which is homogeneous (see Figure 9(C)) and for which connections between adjacent parent vessels are likely to be sustained because the pressure difference is sufficient to maintain an adequate flow in the new vessels. When non-periodic boundary conditions are imposed, vessels that form near the boundary are highly likely to be pruned as they can only form connections to vessels with similar pressures.

### Tumour elimination and its dependence on domain size

**Figure 11:**
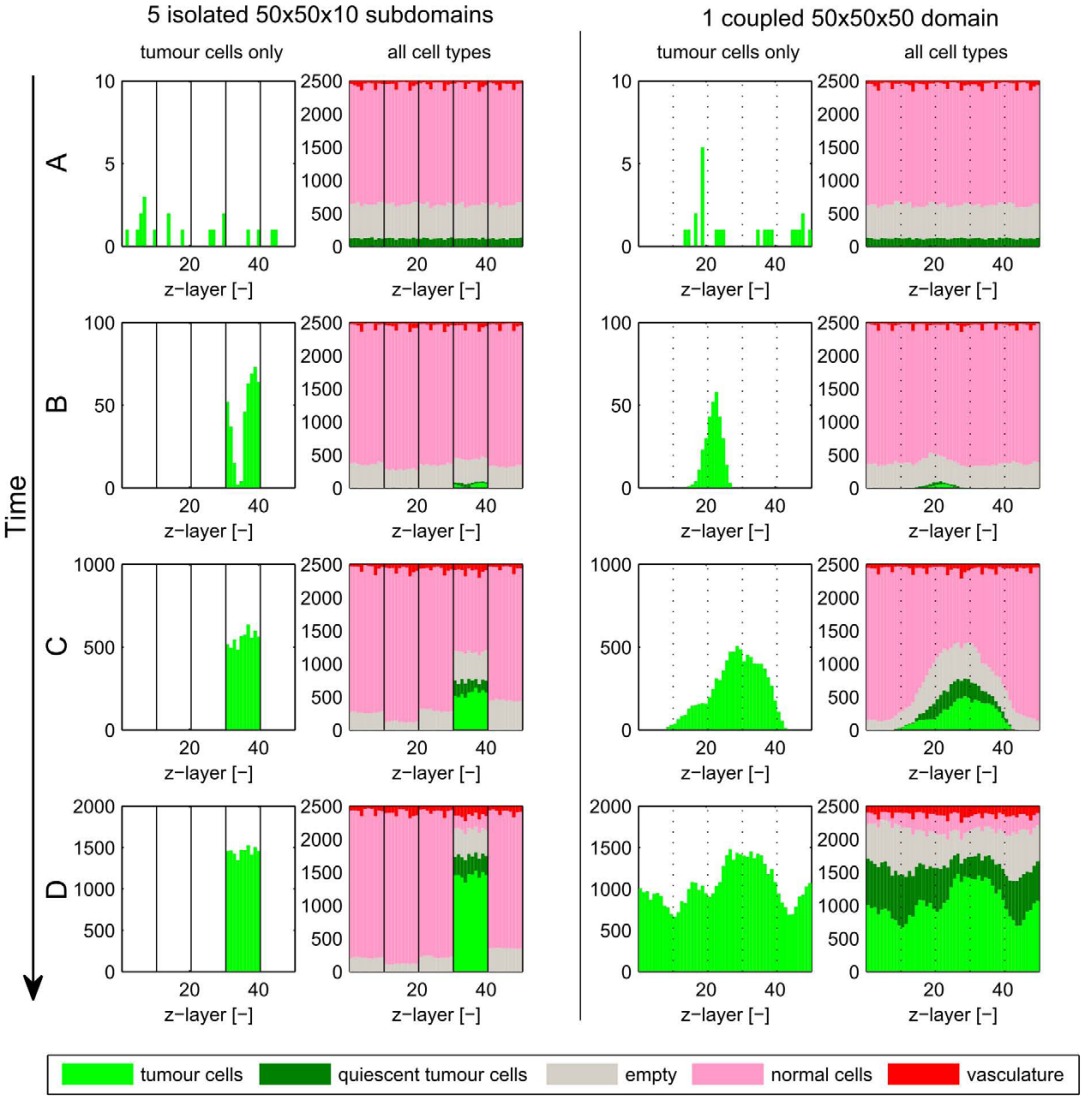
Stochasticity of results and implications on growth, regrowth and therapy. To study the influence of domain size on tumour elimination (e.g. after therapy), we implanted tumour cells in a hostile environment (in which 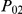 is reduced from 3800 to 3116, hence reducing nutrient delivery to the tissue). Columns 1 and 2 show the number of tumour cells only and the numbers of all cell types respectively, for five independent simulations in an isolated 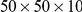-domain. Columns 3 and 4 show equivalent results from one simulation in one large 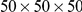 domain. **Row A**) At 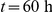 most of the initial tumour cells have died and only a few survived in both cases. At this early stage both isolated and coupled simulations lead to similar results, as the coupled subdomains in the 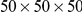 case can be viewed as stochastically independent. **Row B**) At 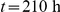 more tumour cells have died and only one isolated subdomain is occupied by tumour cells. The cells are restricted in their motion in the 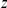-direction in the case of isolated subdomains, whereas the tumour can spread easily in the unrestricted case. **Row C**) At 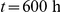 competition between tumour cells and normal cells means that tumour growth slows down – this effect is stronger in the isolated subdomain. **Row D**) At 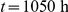 the tumour is eliminated in 4/5 isolated subdomains, but in the larger domain the subdomains are not independent of one another and the tumour has spread into all 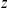-layers. In such a case one overpredicts the efficacy of therapies if one simulates in 2D or in a small three-dimensional subdomain.

The above results demonstrate the influence of domain size on vascular tumour growth. Therefore it is important to assess how the domain size affects predictions about the efficacy of cancer therapies. A key question is whether predictions about tumour response to therapy in larger domains can be extrapolated from simulation results on smaller domains. We investigated these issues by performing the following simulations: a small tumour is implanted in a poorly vascularised tissue with reduced oxygen delivery, such as would arise following exposure to a vascular-targeting agent (e.g. Combretastatin [32]). We model this effect by setting a decreased oxygen permeability coefficient 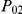. In this case the environment is so hostile that many tumour cells will die, but we seek to determine conditions under which the tumour is completely eliminated, since if one tumour cell survives it would eventually repopulate the entire domain. We illustrate this effect in Figure 11 where we compare the evolution of the tissue in five isolated 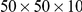 subdomains with that in a single 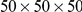 domain. At early times the dynamics in both cases are similar, with small numbers of tumour cells surviving in each subdomain (whether isolated or coupled). While the similarity persists for a short period of time, eventually the tumour in the larger (coupled) domain is able to colonise the entire tissue, whereas the tumour survives in only one out of five isolated subdomains. This is because the small subdomains that comprise the larger simulation are connected, enabling any surviving tumour cells in one subdomain to move into neighbouring subdomains, and to colonise any empty space created by tumour cell death there. When the subdomains are isolated, tumour cells can only expand in the 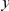-direction and their growth in this direction is inhibited after a small period of time by competition with normal cells.

**Figure 12:**
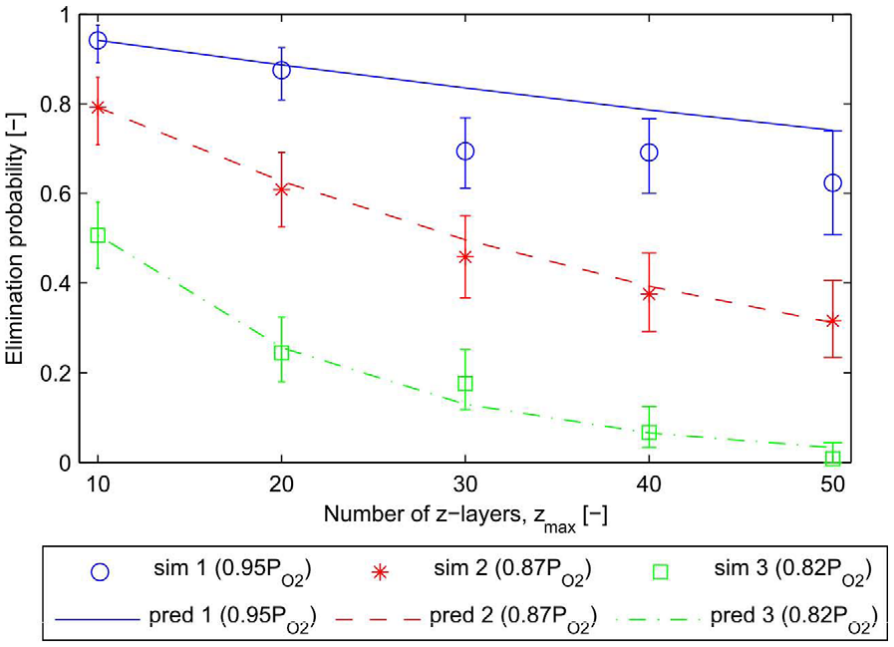
Tumour elimination probability dependency on domain-size. In order to study the influence of domain size when tumour cells are very likely to die (i.e. therapy, implantation), we implanted tumour cells in a hostile environment in which most of the tumour cells die within the first hours post implantation. Increasingly hostile environments (which mimic anti-tumour therapies) are initiated by reducing the oxygen permeability coefficient from 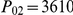 to 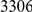, and finally 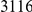. For each value of 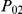 we ran 120 realisations, and the points indicate the estimated mean probability of tumour elimination, 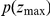, for five domain sizes (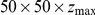). The bars indicate the 95% confidence intervals obtained by boostrapping. If the probability of elimination in each subdomain is independent of the others, then we should have 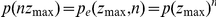. In each case we plot the extrapolated elimination probability based on 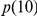. The extrapolated values are in good agreement with the results from direct simulations — with the predicted mean lying within the 95% confidence intervals in most cases.

Motivated by these observations, we estimate the probability of tumour elimination, 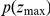, for tissue domains of size 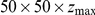 by performing multiple realisations and calculating the frequency of elimination. We denote the number of non-quiescent tumour cells at time 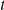 by 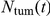. A tumour is said to have been eliminated at time 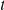 if 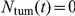, and to have survived if 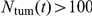 for 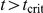, where 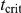 is sufficiently large (several days) to ensure that the influence of the hostile environment can be neglected. This may correspond to a case in which clearance of some therapeutic agent means that its concentration has fallen sufficiently low to be negligible. Figure 12 shows the estimated probability of regrowth for three increasingly severe environments (which we would expect to have increasing elimination probabilities) and a range of domain sizes. If subdomains behave independently then 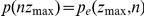 where 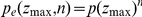 is the extrapolated probability of elimination, i.e. if the domain size is increased by a factor of 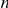 (to have 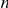 connected subdomains), all the subdomains must remain tumour-free to ensure elimination of the tumour. Figure 12 shows that the extrapolated elimination probabilities are in good agreement with the results from direct simulations — with the predicted mean lying within the 95% confidence intervals in most cases. We conclude that, for the elimination scenario considered here, the subdomains behave as if they are independent of one another, and that predictions about therapeutic efficacy for larger domains can be extrapolated from simulation results for smaller domains.

### Simulations with realistic initial vasculature

This section documents results of a vascular tumour growth simulation for which the initial vascular geometry was taken from multiphoton fluorescence microscopy. The aim here is to integrate the mathematical model with *in vivo* experimental data. To generate experimental data on *in vivo* tumour vasculature, we implanted into a mouse dorsal window chamber a tumour construct comprising a central core of human breast cancer cells surrounded by rat microvessel fragments, embedded in a collagen matrix. The cancer cells and rat microvascular cells express different fluorescent proteins so that, following implantation, the tumour and its vascular network can be visualised. Further details of the experimental methods are presented in Text S1.

**Figure 13:**
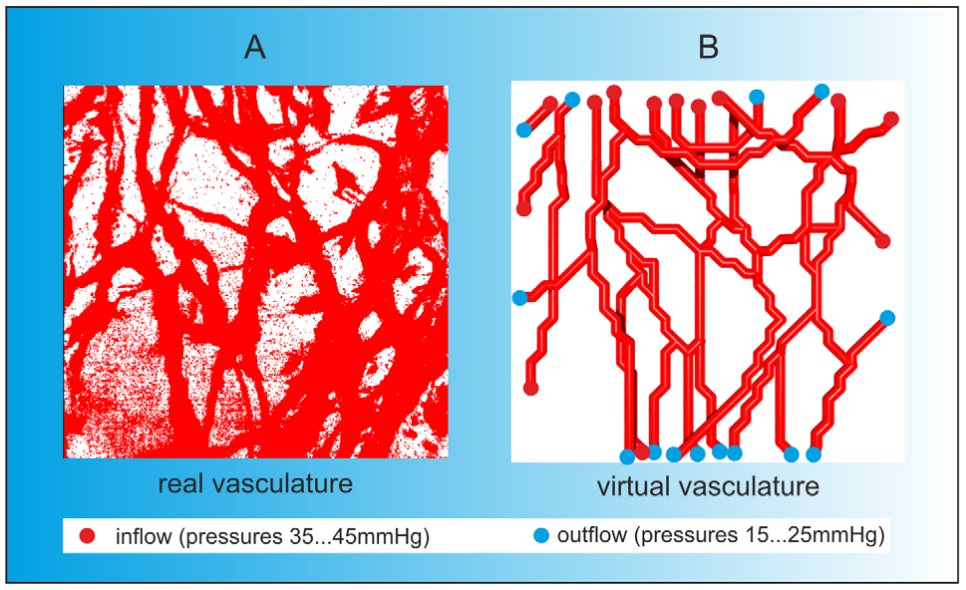
Image reconstruction. We reconstructed the vascular network by applying the following strategy. 3D multiphoton fluorescence microscopy images (A) taken from mouse models *in vivo* formed the basis of our geometrical reconstruction. These images were transferred to OpenInventor and Matlab for image analysis. Based on the data we reconstructed the vascular graph model that describes the connectivity of the vascular network. B) We assigned inflow (red points) and outflow nodes (blue points) at various pressures in order to obtain a persistent and stable network. The vascular graph is characterised by the spatial coordinates of the nodes and the connections between them.

We used the experimental data to reconstruct the vascular graph model, locating nodes in the vessel centres and connecting them by edges, see Figure 13. We use this example principally as proof-of-concept. First, we embed the vascular system into healthy tissue and then simulate vessel adaptation until a steady-state is reached.

**Figure 14:**
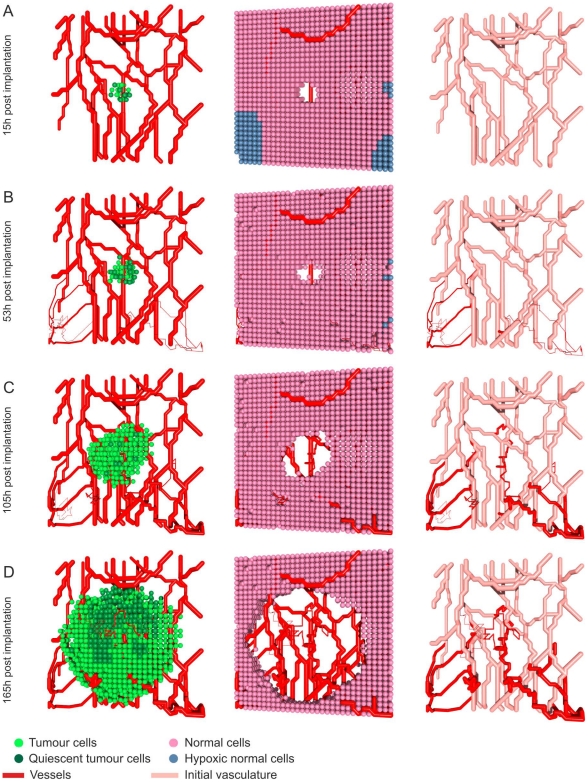
Proof-of-concept: tumour growth in an experimentally derived vascular network. **A**)**–D**) show the temporal evolution of a tumour in a real vascular network embedded in normal tissue (see Video S3). As initial condition we have taken a vascular network from multiphoton fluorescence microscopy and embedded it in a 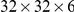 cellular automaton domain. In the first column the tumour expands radially, and degrades the healthy tissue (second column). The predicted adaptations of the vascular system are shown in the third column where the experimentally derived network is shown in light red, while the new vessels are coloured in red.

Currently in the models the vasculature is embedded in a healthy tissue into which a small tumour is implanted and its evolution is studied. A projection of a 3D image set of the tissue is presented in Figure 13. In Figure 14 we observe that the tumour expands radially into the surrounding healthy tissue which is degraded by the cancer cells by decreasing the p53 death-threshold for normal cells. Normal cells in the lower left and right corners of the simulation domain (first column) are exposed to low oxygen (hypoxia), and hence produce VEGF which induces an angiogenic response in our model. While the new vessel in the lower left corner is persistent and increases in radius, the vessel in the lower right corner is pruned back. In this case pruning occurs because the new blood vessel connects vessels from the initial network that have similar pressures. In general it can be said that the normal cells are adequately nourished by oxygen as only a few hypoxic cells can be observed in simulations with normal cells only. In contrast, we find a high percentage of quiescent cancer cells in all states of tumour growth, leading to further angiogenesis in our simulations (see Figure 6). The dark red vessels in row 3 indicate new vessels that develop after tumour implantation. In conclusion, our model predicts an increase in the vascular density following tumour implantation.

**Figure 15:**
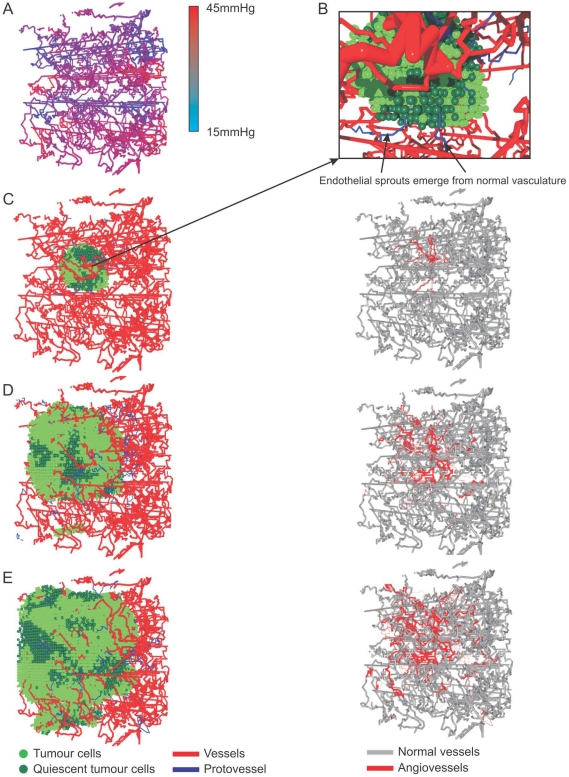
Tumour angiogenesis with enhanced chemotaxis. We increased the endothelial tip cell chemotaxis coefficient by a factor of 10 (so that 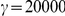), and generated a “normal” vascular network from the parent vessel configuration in Figure 9(C) (i.e. with doubly-periodic boundary conditions) by filling the domain with normal cells and allowing angiogenesis and vessel remodelling to proceed (Video S4 and Video S5). We then implanted a small tumour into the new “normal” network. The tumour stimulates further sprouting, so that the tumour vasculature is more dense than the corresponding normal network. **A**) The generated vascular network and pressure distribution for normal tissue. **B**) A short time after implantation the first endothelial sprouts appear and migrate into the tumour. The appearance of new vessels within the healthy tissue around a tumour is an effect that can often be observed in gliomas. **C**)**–E**) A time-series of the growing tumour in the normal vasculature, the normal cells that surround the tumour are faded out (Video S6). On the right hand side the changes to the normal vasculature due to the implanted tumour are shown. The original normal vasculature is depicted in grey whereas new vessels that emerge due to the angiogenic response are coloured red (Video S7). In D) one can see an effect of the doubly periodic boundary conditions where tumour cells that departed through the upper boundary enter again via the lower boundary.

Finally, in Figure 15 we present results from a simulation for which the chemotactic sensitivity, 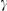, of endothelial tip cells is increased by a factor of 10, so that 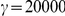, and the doubly-periodic parent vessel geometry described in Figure 9(C) is imposed. We generated a “normal” vascular network from these parent vessels by filling the domain with normal cells and allowing angiogenesis and vessel remodelling to proceed. Since endothelial tip cell movement is more strongly directed than in the previous simulations, the resulting vasculature is less tortuous and less dense than in previous examples (compare Figures 3 and 15). Figure 15 shows what happens when a small tumour is implanted into the new “normal” network. The tumour stimulates further capillary tip sprouting, and generates a tumour vasculature which is more dense than the corresponding normal network.

## Discussion

In this paper we have extended an existing multiscale model of vascular tumour growth from 2D to 3D. By performing extensive numerical simulations we have investigated the ways in which the emergent system dynamics are influenced by the 3D formulation. Starting from the idealised case of growth from a single pair of parent vessels in a 2D domain we found that the vessel volume fraction decreases as the 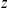-extent is increased—each vessel can adequately perfuse a small number of cell layers without expansion of the vasculature. However, as the 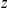-extent increases beyond a characteristic length scale set by the transport and uptake of oxygen, the vessel volume fraction rises again as more cells at a distance from vessels become hypoxic and stimulate a compensatory angiogenic response.

We then considered whether it is possible to represent a large three-dimensional tissue domain by smaller subdomains, and whether the ability to do this depends on the choice of boundary conditions and the initial distribution of blood vessels. For a large domain, with many pairs of parallel vessels, two subdomains (two vessel pairs) give a reasonable representation of the dynamics in the larger domain, but a single subdomain can generate quite different characteristics. Even with periodic boundary conditions, one vessel pair is not representative of two (or more). This is in part because, although periodic boundary conditions allow vessel connections to be made from a parent vessel to itself (by wrapping round in the 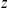-direction), it is not possible for one parent vessel to sprout in both directions from the same or adjacent points (since we impose an exclusion distance around each sprout within which no other sprouts may emerge). In contrast, in a perfectly symmetric scenario with two (or more) separate parent vessels, each parent vessel would sprout at the same place, so that, on average, there would be connections between parent vessels at the same pressure (which would therefore be pruned). Thus we expect more pruning in the larger domain than in the smaller domain, even with periodic boundary conditions. We remark that in a deterministic partial differential equation model we could guarantee identical solutions provided the initial conditions were equivalent. We also found that the long-time tumour and vessel fractions were almost identical for the small domain with the three choices of boundary conditions, although tumour growth was fastest in the doubly-periodic case (see Figure 8). This can be understood by noting that in the doubly-periodic case vessel connections can form more rapidly, promoting faster tumour growth.

We remark that it is, in principle, possible to impose triply-periodic boundary conditions that allow vessels and cells to leave and re-enter the domain in all spatial directions. A problem that arises with triply periodic boundary conditions relates to the pressure drop that is applied to the initial vascular network. The vessel radii adapt to several different stimuli (see the model section). Imposing periodicity in the axial direction of the initial vessels would demand that the vessel radii at both ends of the domain be equal, which is not usually the case. Pressure and flow calculations are also problematic: one has to keep track of the boundary across which vessels leave and re-enter the domain. The total pressure drop along each vessel is similar in a periodic alignment, whereas the absolute pressures differ. If a sprout from close to a low-pressure outflow leaves the domain in a downstream direction it re-enters at the opposite domain side and can connect to the original network close to a high-pressure inflow. This would lead to a very short vessel segment with a nearly maximal pressure drop, which could lead to extreme vessel dilation. The same principle holds for vessels that leave the domain in the upstream direction. One potential resolution to this problem would be to consider only the pressure drop 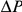 across the basic unit of a triply periodic domain, adding or subtracting multiples of 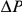 at certain nodes to account for the number of times connecting vessels wrap around the domain. However, the dependence of vascular adaptation (and other features such as arterio-venous identity) on absolute pressures makes this impractical.

Building on these results for a basic subdomain with two parallel countercurrent initial vessels, we showed that the choice of basic repeating unit can have a significant impact on the system's dynamics. In particular, for a unit of eight parallel vessels with equal pressure drops, but a variety of mean pressures, we found that a single unit was representative of larger domains. In these cases, the choice of boundary conditions had a strong influence on the system's dynamics. For doubly-periodic boundary conditions the tissue grows in an efficient manner, with a low vascular density—this is because the initial vessels are in close proximity and their mean pressures are sufficiently different (as illustrated in Figure 9(C)). For non-periodic and singly-periodic boundary conditions, vessels near the reflecting boundaries can only connect to vessels with similar pressures, so that there is more pruning and the vasculature is less efficient.

The flexibility of the simulation domain is expected to play a significant role in tumour response to therapy. We simulated a nutrient-deprived environment, such as might arise from vascular-regression therapies, and found that predictions for therapeutic efficacy for larger domains could be inferred from simulations on smaller domains. We speculate that the ability to extrapolate predictions of tumour elimination to larger domains could be extended to other forms of therapy, e.g. cytotoxic drugs and radiotherapy. We anticipate that this may be dependent on the details of particular therapies, in particular on the timescale and spatial extent of their action. Such investigations could form the basis for future research.

As proof-of-concept, we then used an experimentally-derived vessel network to initialise a simulation of tumour growth and angiogenesis. To the best of our knowledge, this is the first time this has been done—Secomb, Pries and co-workers (e.g. [5], [6]) have used such networks to study structural adaptation alone. It paves the way for further research which will make a closer link with experimental data. In particular, it would be of great interest to use experimental data with two or more time points, use the first time point to initialise simulations, and then compare the simulation with data at later time points. We would not expect to obtain a detailed match at later time points, since we simulate a stochastic system, but we would expect agreement between experimental and simulated values for certain characteristics, such as vessel volume fractions and the distributions of vessel radii and segment lengths.

In summary, we have shown that a small subdomain has to have a certain size and a certain characteristic initial vessel structure to ensure that it represents larger domains. This has important implications for modelling therapy, and raises crucial questions about how to use multiscale models grounded at the cellular-level to inform modelling and our understanding at the scale of tissues or whole tumours.

## Supporting Information

Video S1 Simulation showing the dynamics of cancer cells and the vascular system with two straight vessels as initial condition which correspond to Figure 3.
(3.34 MB WMV)

Video S2 Simulation showing the dynamics of normal cells and the vascular system with two straight vessels as initial condition which correspond to Figure 3.
(4.09 MB WMV)

Video S3 Simulation showing tumour evolution with an experimentally-derived initial vascular network, illustrating the steps depicted in Figure 14.
(5.59 MB AVI)

Video S4 Simulation showing the development of a vascular system with increased endothelial cell chemotaxis, with the normal cells faded out. (Results in Figure 15(A)).
(1.12 MB WMV)

Video S5 Simulation showing the evolution of a vascular system, with 16 straight initial vessels, embedded in a population of normal cells. Increased endothelial tip cell chemotaxis leads to the vascular system depicted in Figure 15(A).
(2.29 MB WMV)

Video S6 Simulation showing tumour cells growing in a normal vascular network and additionally inducing angiogenesis (Figure 15, column 1 of (C)–(E)).
(1.71 MB WMV)

Video S7 Simulation showing the angiogenic response of the vascular system when a tumour is implanted. The normal vascular network is coloured in grey, whereas new angiogenic vessels are coloured red (Figure 15, column 2 of (C)–(E)).
(0.96 MB WMV)

Text S1 Mathematical model, experimental methods and tables. A more detailed description of the mathematical model, experimental methods and the parameter values are given in this supporting information file.
(0.25 MB PDF)
