# Supplementary material for: Multiscale Modelling of Vascular Tumour Growth in 3D: The Roles of Domain Size and Boundary Conditions
Source: PLoS One. 2011 Apr 13;6(4):e14790. doi: 10.1371/journal.pone.0014790 (PMC3076378; doi:10.1371/journal.pone.0014790)
Supplement: Text S1 — Mathematical model, experimental methods and tables. A more detailed description of the mathematical model, experimental methods and the parameter values are given in this supporting information file. (0.25 MB PDF) [file pone.0014790.s008.pdf]

Multiscale modelling of vascular tumour growth in 3D: the roles of domain size and boundary conditions

Holger Perfahl[1,*], Helen M. Byrne[2], Tingan Chen[6], Veronica Estrella[6], Tomás Alarcón[3], Alexei Lapin[1], Robert A. Gatenby[6], Robert J. Gillies[6], Mark C. Lloyd[6], Philip K. Maini[4,5], Matthias Reuss[1], Markus R. Owen[2]

Mathematical model

A more detailed description of the mathematical model is presented in this appendix. Our description starts at the smallest spatial scale, namely the subcellular layer. Then the cellular and diffusible layers are introduced, and finally the vascular layer is introduced. Interactions between these layers are highlighted in the final part of this section where the computational algorithm is presented. The parameter values are listed in Tables (1)-(4).

Subcellular layer

Coupled systems of nonlinear ordinary differential equations (ODEs) are used to model progress through the cell cycle, and changes in expression levels of p53 and VEGF [1]. In practice the cell cycle can be divided into 4 phases: during G_1 the cell is not committed to replication, but if conditions are favourable it may enter the S (synthesis) phase, in which DNA replication takes place. During the G_2 phase, further growth, and DNA and chromatid alignment occurs, before the cell divides during M (mitosis) phase. For the cell cycle, we consider the cell mass M and the proteins cycCDK (cyclin-CDK complex), Cdh1 (Cdh1-APC complex), p27 and npRB (non-phoshorylated retinoblastoma protein). The cell cycle model that we consider focuses on the G_1-S transition. It extends an earlier model due to Tyson and Novak [2] by accounting for the p27-mediated effect that hypoxia has on the cell cycle [3]. Using square brackets

to represent intracellular protein concentrations we have

$$\frac{\mathrm{d}[\mathrm{Cdh1}]}{\mathrm{d}t} = \frac{(1+b_3[\mathrm{npRB}])(1-[\mathrm{Cdh1}])}{J_3+1-[\mathrm{Cdh1}]} - \frac{b_4 M[\mathrm{cycCDK}][\mathrm{Cdh1}]}{J_4+[\mathrm{Cdh1}]} \tag{1}$$

$$\frac{\mathrm{d}[\mathrm{cycCDK}]}{\mathrm{d}t} = a_4 - (a_1 + a_2[\mathrm{Cdh1}] + a_3[\mathrm{p27}])[\mathrm{cycCDK}] \tag{2}$$

$$\frac{\mathrm{d}M}{\mathrm{d}t} = \eta M\left(1-\frac{M}{M^*}\right) \tag{3}$$

$$\frac{\mathrm{d}[\mathrm{p27}]}{\mathrm{d}t} = c_1\left(1-\chi\frac{M}{M^*}\right) - \frac{c_2 c_{02}}{B+c_{02}}[\mathrm{p27}] \tag{4}$$

$$\frac{\mathrm{d}[\mathrm{npRB}]}{\mathrm{d}t} = d_2 - (d_2 + d_1[\mathrm{cycCDK}])[\mathrm{npRB}]; \tag{5}$$

where b_3, J_3, b_4, J_4, a_1, a_2, a_3, a_4, η, M^*, c_1, c_2, B, χ, d_1 and d_2 are constants, specified in Table (1).

In equations (1)-(5), where M is small, the cell is maintained in a state corresponding to G_1 for which levels of Cdh1 are high and levels of cycCDK are low. Growth in the cell mass increases Cdh1 degradation and reduces p27 production, so that cycCDK increases. This leads to inhibition of npRB and Cdh1 and, hence, positive feedback on cycCDK. At a certain point, corresponding to the G_1-S transition, the state with high Cdh1 and low CDK is lost, and a state with low Cdh1 and high cycCDK is attained. Finally, when Cdh1 levels are sufficiently low and CDK levels sufficiently high, cell division occurs. The external O_2 concentration c_{02} couples the subcellular and diffusible scales by influencing progress through the cell cycle. Decreasing c_{02} reduces p27 degradation, and the resulting increase in levels of p27 counteracts the effect of the increasing mass on cycCDK. In particular if c_{02} levels are sufficiently the G_1-S transition cannot occur. Further details about the model can be found in [1, 3].

The intracellular concentration of p53 regulates normal cell apoptosis, and that of VEGF controls VEGF release by normal cells. The dynamics of p53 and VEGF are coupled to one-another, and to the extracellular oxygen concentration, as described by the following differential equations

$$\frac{\mathrm{d}[\mathrm{p53}]}{\mathrm{d}t} = k_7 - k_7'\frac{c_{02}}{K_{\mathrm{p53}}+c_{02}}[\mathrm{p53}], \tag{6}$$

$$\frac{\mathrm{d}[\mathrm{VEGF}]}{\mathrm{d}t} = k_8 + k_8''\frac{[\mathrm{p53}][\mathrm{VEGF}]}{J_5+[\mathrm{VEGF}]} - k_8'\frac{c_{02}}{K_{\mathrm{VEGF}}+c_{02}}[\mathrm{VEGF}], \tag{7}$$

with the constants k_7, k_7', K_{p53}, k_8, k_8', k_8'', J_5 and K_{VEGF} (see Table (1)). The ordinary differential equations (1)-(7) are solved subject to the initial conditions specified in Table (1), using the open source CVODE library (`https://computation.llnl.gov/casc/sundials/main.html`).

Cell death, quiescence and proliferation are determined by a cell's internal protein concentrations. We apply the following rules to identify the different cell-states. In normal cells, cell death occurs if $[\mathrm{p53}] > \mathrm{p53}_{\mathrm{THR}}$, where $\mathrm{p53}_{\mathrm{THR}}$ is the maximal threshold they can sustain before committing apoptosis, and is given by

$$\mathrm{p53}_{\mathrm{THR}} = \begin{cases} \mathrm{p53}_{\mathrm{THR}}^{\mathrm{high}} \text{ for } \rho_{\mathrm{normal}} > \rho_{\mathrm{THR}} \\ \mathrm{p53}_{\mathrm{THR}}^{\mathrm{low}} \text{ for } \rho_{\mathrm{normal}} \le \rho_{\mathrm{THR}} \end{cases}. \tag{8}$$

We define the set of cells in the neighbourhood of cell i as Θ_i. The normal cell ratio in Equation (8) is given by

$$\rho_{\mathrm{normal}}(i) = \frac{\sum_{k\in\Theta_i} \text{normal cells at site } k}{\sum_{k\in\Theta_i} \text{normal or cancer cells at site } k}. \tag{9}$$

Definition (9) accounts for the fact that healthy cells are more likely to die if they live in a tumour environment. This can be caused by the altered microenvironment in tumours. Tumour cells enter quiescence if $[\mathrm{p27}] > \mathrm{p27}_e$, or leave quiescence if $[\mathrm{p27}] < \mathrm{p27}_l$. If a cancer cell is in quiescence for too long ($> T_{\mathrm{death}}$), the cell dies. It should be noted that cancer cell death is not influenced by p53.

The condition to be satisfied for the proliferation of cells is

$$[\mathrm{Cdh1}] < \mathrm{Cdh1}_{\mathrm{THR}} \text{ and } [\mathrm{cycCDK}] > \mathrm{cycCDK}_{\mathrm{THR}}. \tag{10}$$

The daughter cell is placed in the current location if there is free space; otherwise it is moved to an empty neighbour location with a high oxygen concentration. If there is no free space in the neighbour CA-cells, the parent cell dies and no daughter is produced.

Cellular layer

The following section focuses on the creation and movement of new vessels. For a detailed description see Owen et al. [4]. New sprouts form at site i (which must be a vessel site) with probability $\mathcal{P}_i^{\mathrm{sprout}}$ where

$$\mathcal{P}_i^{\mathrm{sprout}} = \frac{P_{\mathrm{max}}^{\mathrm{sprout}} c_{\mathrm{VEGF}}}{V_{\mathrm{sprout}} + c_{\mathrm{VEGF}}} \Delta t. \tag{11}$$

Since VEGF stimulates sprout formation the probability of sprouting is assumed to increase with the VEGF concentration, c_{VEGF}. The maximum sprouting probability is $P_{\mathrm{max}}^{\mathrm{sprout}}$ and V_{sprout} is a constant.

New sprouts can only emerge if sufficient space is available. Around the base of each sprout a radius of exclusion is defined, in which new sprouts cannot occur. For the vessel tip cells, we define $\mathcal{P}(i \to j)$ as the probability of moving from i to j, to be

$$\mathcal{P}(i \to j) = \frac{\Delta t D}{d_{ij}^2 \Delta x^2} \frac{(N_m - N_j)\left(1 + \gamma \frac{c_{\mathrm{VEGF},j} - c_{\mathrm{VEGF},i}}{d_{ij}\Delta x}\right)}{\sum_{k\in\Omega_i}(N_m - N_k) + N_m - N_i + N_m M_c} \tag{12}$$

for $i \neq j \in \Omega_i$. Herein, D represents the cell motility, N_m is the maximal carrying capacity of the cell type attempting to move, N_i is the number of cells, M_c is a constant and $c_{\mathrm{VEGF},i}$ the VEGF level at site i, γ is the chemotactic sensitivity, and Ω_i is the set of sites in the neighbourhood of i, not including i itself. The probabilities are weighted with the distance between lattice site i and j, written as d_{ij}. In the three dimensional case, Ω_i has at most 26 neighbour elements for each lattice point i. We set the probability to zero if an endothelial cell crosses a vessel. The probability of not moving is

$$\mathcal{P}(i \to i) = 1 - \sum_{\substack{j,k\in\Omega_i \\ j\neq k}} \mathcal{P}(j \to k). \tag{13}$$

Whenever a tip cell moves to another location, an endothelial cell remains at the former lattice site. This is equivalent to the snail-trail concept also applied in [5–7]. A sprout dies if it does not connect to another sprout or the existing vasculature within a certain time period.

Diffusible layer

The diffusible layer facilitates the coupling between the vascular and subcellular layers. We consider two diffusible components in our model, namely oxygen and VEGF, and denote their concentrations by c_{VEGF} and c_{02}. The vascular system acts as an oxygen source, while the normal and tumour cells act as sinks. This behaviour is described by the following, quasi-stationary, reaction-diffusion equation

$$D_{02}\Delta c_{02} + 2\pi \tilde{R}(t, \boldsymbol{x}) P_{02}(c_{02}^{\mathrm{blood}} - c_{02}) - k_{02}(t, \boldsymbol{x}) c_{02} = 0. \tag{14}$$

In Equation (14) the vessel radius indicator function $\tilde{R}$ returns the vessel radius if a vessel is present at position $\boldsymbol{x}$ and zero otherwise. Equation (14) also accounts for the vessel permeability to oxygen (P_{02}), the blood oxygen concentration c_{02}^{blood} and the cell type dependent oxygen consumption rate k_{02}. If cells

become hypoxic or quiescent, they start to secrete VEGF, which then can be removed by the vasculature. The concentration of VEGF is determined by

$$D_{\text{VEGF}}\Delta c_{\text{VEGF}} - 2\pi\tilde{R}(t,\boldsymbol{x})P_{\text{VEGF}}c_{\text{VEGF}} + k_{\text{VEGF}}(t,\boldsymbol{x}) - \delta_{\text{VEGF}}c_{\text{VEGF}} = 0, \tag{15}$$

wherein P_{VEGF} is the permeability of the vessels to VEGF, k_{VEGF} the cell type dependent VEGF production rate and the decay rate δ_{VEGF}. In our numerical algorithm, Equations (14) and (15) are discretised with a finite difference scheme and the resulting sparse linear system of equations is solved with a GMRES-solver.

In case of a non-periodic simulation domain it is assumed that there is no flux of diffusible substances over the boundary and thus homogeneous Neumann boundary conditions are imposed. For simulations in a periodic domain we apply periodic boundary conditions for the calculation of diffusible substance concentrations.

Vascular layer

We follow very closely the work of Secomb et al. and refer the reader to [8] for full details. We assume a laminar Poiseuille flow in each vessel. The flux $\dot{Q}_i$ through vessel i is given by

$$\dot{Q}_i = \frac{\pi R_i^4}{8\mu(R_i,H_i)L_i}\Delta P_i, \tag{16}$$

where ΔP_i is the pressure difference at the vessel segment i, L_i the vessel length, $\mu(R_i,H_i)$ is the radius R_i and haematocrit H_i dependent blood viscosity [8]. In Equation (16) we can identify the resistance of vessel i by $\text{Res}_i = 8\mu(R_i,H_i)L_i/(\pi R_i^4)$. In (16) the blood viscosity is defined by

$$\mu(R,H) = \mu_0\mu_{\text{rel}}(R,H), \tag{17}$$

where μ_0 is a positive constant,

$$\mu_{\text{rel}}(R,H) = \left[1 + (\mu_{0.45}(R)-1)\frac{(1-H)^C-1}{(1-0.45)^C-1}\left(\frac{2R}{2R-1.1}\right)^2\right]\left(\frac{2R}{2R-1.1}\right)^2, \tag{18}$$

$$\mu_{0.45}(R) = 6e^{-0.17R} + 3.2 - 2.44e^{-0.06(2R)^{0.645}} \tag{19}$$

and

$$C = C(R) = \left(0.8 + e^{-0.15R}\right)\left(-1 + \frac{1}{1 + 10^{-11}(2R)^{12}}\right) + \frac{1}{1 + 10^{-11}(2R)^{12}}. \tag{20}$$

Using Equations (16)-(20) we can calculate the flux through each vessel segment in terms of the pressure at each junction of the vascular tree. At any node of the vascular network, the total flow into that node must balance the total flow out of that node. With the pressures at each inlet and outlet (P_{in} and P_{out}) prescribed, we obtain a linear system of equations for the pressures at each vessel node. This system is solved with the direct SuperLU solver.

When updating the vascular network, there are two different timescales of interest, the timescale for flow and the timescale for vascular adaptation. While changes in flow may be rapid, we assume that vascular adaptation occurs on the same timescale as endothelial cell movement and cell division. Consequently we model the temporal evolution of a vessel segment's radius by applying the following, discretised ordinary differential equation

$$R(t + \Delta t) = R(t) + \alpha_R \Delta t R(t)(S_h + S_m - k_s), \tag{21}$$

where Δt is the timestep size and the updated radius must satisfy the constraint $R_{\min} \leq R(t+\Delta t) \leq R_{\max}$. The factor α_R that appears in Equation (21) relates the stimuli to our timestep size Δt. In the absence of any details on the rate of vascular adaptation (since all previous studies that we are aware of consider quasi-steady state vessel radii), we set $\alpha_R = 3.3 \times 10^{-6}\text{min}^{-1}$ so that the rate of change of the vessel radius is typically less than 10% per hour. k_s is the shrinking tendency of a vessel that takes into account that vessels tend to regress in the absence of stimuli. S_h and S_m are haemodynamic and metabolic stimuli for vascular adaptation:

- **Haemodynamic stimulus:**

$$S_h = \log(\tau_w + \tau_{\text{ref}}) - k_p \log((\tau(P)), \tag{22}$$

with the wall shear stress (WSS)

$$\tau_w = \frac{R\Delta P}{L}, \tag{23}$$

the constant reference wall shear stress τ_{ref} and the corresponding set point pressure of the wall

shear stress $\tau(P)$, described by the empirical function

$$\tau(P) = 100 - 86 \exp\left(-5000 \left[\log(\log P)\right]^{5.4}\right). \tag{24}$$

- **Metabolic stimulus:**

  $$S_m = k_m(c_{\mathrm{VEGF}}) \log\left(\frac{\dot{Q}_{\mathrm{ref}}}{\dot{Q}H + a\dot{Q}_{\mathrm{ref}}} + 1\right), \tag{25}$$

  with

  $$k_m(c_{\mathrm{VEGF}}) = k_m^0 \left(1 + k_m^{\mathrm{VEGF}} \frac{c_{\mathrm{VEGF}}}{V_0 + c_{\mathrm{VEGF}}}\right), \tag{26}$$

  where $\dot{Q}_{\mathrm{ref}}$, a, k_m^0, k_m^{VEGF} and V_0 are parameters. $\dot{Q}_{\mathrm{ref}}$ is a reference flow, and the term $a\dot{Q}_{\mathrm{ref}}$ (where a is a small parameter) in the denominator of Equation (25) is introduced to avoid extreme vessel dilation in poorly perfused vessels (and hence S_m differs slightly from the original model [4, 9]).

In addition to being created new vessels can also be removed by pruning. If a vessel is exposed to a wall shear stress that is below a threshold ($\tau_{\mathrm{w}}^{\mathrm{crit}}$) for a certain time ($T_{\mathrm{prune}}$), we remove the vessel from our system. In general the vascular adaptation algorithm includes the following steps. First, the vessel radii evolve according to Equation (21). Then we calculate the flows in the network. In contrast to the previous model [4], the evolution of the radius is considered separately and is not iterated until a steady-state is reached.

Computational algorithm

The basis of our model is a regular grid that subdivides the simulation domain into cellular automaton lattice sites. Each lattice site can be occupied by several biological cells and vessels. Figure 1 shows the high degree of coupling between the different spatial scales. We introduce the main steps in the following list. The flowchart in Figure 2 summarises the overall algorithm and the temporal order of computation, and shows how processes that act of different space and time scales are inter-related.

1. **Initialisation** *(Vascular and cellular layers)*

   We specify an initial vascular network as a system of straight pipes with fixed inflow and outflow nodes with prescribed pressures. We also prescribe the amount of haematocrit that enters through each inlet, and the initial location of the different cell types in the cellular automaton domain.

2. **Update cells, oxygen and VEGF** *(Diffusible, cellular and subcellular layers)*

   - **Calculation of oxygen concentration** *(Diffusible layer)*

     The reaction-diffusion equation (14) is used to calculate the quasi-stationary oxygen concentration $c_{02}(t, \boldsymbol{x})$ in the simulation domain. Oxygen consumption by normal and cancer cells is included in Equation (14) via sink terms, assuming first order kinetics. On the other hand, perfused blood vessels deliver oxygen to the tissue and thus account for oxygen sources.

   - **Calculation of cell cycle, p53 and VEGF ODEs** *(Subcellular layer)*

     The subcellular level is coupled to its local environment via the oxygen concentration. Oxygen drives the cell cycle of individual cells, whose current state is described by the time-dependent concentrations of the proteins $\mathrm{Cdh1}, \mathrm{cycCDK}, \mathrm{p27}, \mathrm{npRB}$ and the cell mass M. Internal p53 and VEGF concentrations are also considered. All subcellular variables are modelled by the coupled systems of non-linear ordinary differential equations (1)-(7).

   - **Check for cell division** *(Cellular layer)*

     Cells divide if their Cdh1 and cycCDK concentrations are under, respectively, over a predefined threshold (see Equation (10)).

   - **Cell movement** *(Cellular layer)*

     Vascular tip cells perform a biased random walk through the tissue. The probability of moving in a certain direction is influenced by the local VEGF gradient and cell densities (see Equation (12)). The motility of normal and cancer cells is also included via Equation (12).

   - **Calculation of VEGF concentration** *(Diffusible layer)*

     Quiescent tumour cells and hypoxic normal cells produce VEGF, and so contribute to the source term in the reaction-diffusion equation (15) for the VEGF concentration $c_{\mathrm{VEGF}}(t, \boldsymbol{x})$. VEGF is removed by the vascular system.

   - **Check for cell quiescence** *(Cellular layer)*

     Tumour cells enter or leave a quiescent state depending on the internal cell p27 concentration, which is described in Equation (4). Oxygen is the external factor that influences the level of p27.

   - **Check for cell death** *(Cellular layer)*

     Normal cells die if their subcellular p53 concentration exceeds a threshold value. If a normal

cell is surrounded by a high number of tumour cells then its p53 threshold for cell death is reduced (see Equation (8)). This models the degradation of a tumour's environment by tumour cells. Tumour cells die if they are quiescent for a certain period of time; unlike normal cells, their death is not influenced by p53.

3. **Update vasculature** *(Cellular and vascular layer)*

   The vascular system continually remodels and evolves in response to external and internal stimuli.

   - **Check for new tip cells** *(Cellular layer)*

     A raised VEGF concentration in the tissue stimulates the vasculature to form new sprouts. The probability that new sprouts emerge is specified by Equation (11) and is an increasing function of the local extracellular VEGF concentration.

   - **Check for anastomosis** *(Cellular layer)*

     New vessels are formed if sprouts connect to other sprouts or to the pre-existing vascular network.

   - **Vessel pruning** *(Vascular layer)*

     Vessels that are underperfused ($\tau_{\mathrm{w}} < \tau_{\mathrm{w}}^{\mathrm{crit}}$) for a certain period of time (T_{prune}) are removed from the vascular network.

   - **Calculation of radius adaptation** *(Vascular layer)*

     The vessel radii are updated at each timestep according to equation (21). The change in vessel radii is influenced by haemodynamic and metabolic stimuli as well as the general tendency of vessels to shrink (see Equations (22)-(25)).

   - **Calculation of pressures and flows within vasculature** *(Vascular layer)*

     The pressure at each node of our vascular network is calculated by applying conservation of mass at each node, where the fluxes are assumed to obey Poiseuille's law (16), and the haematocrit is assumed to split symmetrically at bifurcations.

In 1) the whole cellular automaton model is initialized. Then 2) and 3) are carried out within each time interval Δt until the final simulation time is reached.

Experimental methods

GFP-expressing microvessel fragments are isolated from the epididymal fat pads of GFP/rats which are genetically engineered to express GFP (RRRC Rat Resource and Research Center; Columbia, MO, USA) and the epididymal fat is isolated from rats that are greater than 300g. The fat pads are aseptically removed, minced and digested in DNase (1mg/ml) and collagenase (2mg/ml). Following digestion, 2 filtration steps take place. First a 500μM filter is used to remove large tissue debris and then a 20μM filter is used to retain the microvessel fragments while removing smaller debris such as cells. The microvessel fragments are then added to a solution of Type I rat tail collagen (BD Biosciences) and DMEM. The final concentration of rat tail collagen that is used is 3mg/ml, the DMEM is 1X and the concentration of fragments is $15,000 - 20,000$ fragments/ml.

The tumour droplet consists of the MDA MB 231/RFP cells that will form the tumour. The cells are used in a final concentration of 12.5×10^6/ml and are suspended in 2.5mg/ml of rat tail Type I Collagen (BD Biosciences) and 1X DMEM. Using a 48 well non-tissue cultured plate, a 15 μl drop of the tumour cell suspension is polymerized in the center of the well. Following brief polymerization of about 2 minutes at $37°C$ in the incubator, 200μl of the microvessel fragment mix is added to the wells creating a donut shape around the tumour cell droplet. This tumour construct was then placed in the $37°C$ incubator for 20 minutes. Following polymerization, 250μl of 1X DMEM (+10% FBS) was added to the tumour construct and incubated at $37°C$ for 4 days. Within these 4 days the microvessels undergo spontaneous angiogenesis and anastamosis. Following the 4 day incubation the tumour construct is inoculated into the dorsal window chamber.

Dorsal window chamber systems are made of titanium steel and are sterilely implanted on the dorsal side of a $8 - 10$ weeks old NOD/SCID mouse. The chamber holds a flap of dorsal skin up vertically away from the mouse's body. A small layer of skin is surgically excised from the flap creating a pocket. Droplets containing RFP-expressing MDA-MD 231 breast cancer cells and GFP-expressing rat microvessel cells are inoculated on to the region of exposed tissue. A #1.5 1cm diameter round glass cover-slip is placed over the tissue containing the implanted tumour and microvessel mix and seals the wound. This cover slip is located in the window portion of the chamber.

In order to image the tumour cells and vascular network within the window chamber, the mice are anesthetised and fitted with a nose cone affixed to a portable inhalation isofluorane cart. A customized

fluorinated ethylene propylene mount is used to fix the mouse on the stage of the microscope which keeps the mouse in place and minimizes cardiac, pulmonary or other movements. Images are taken using the Olympus FV1000MPE multiphoton excitation microscope with a combination of objectives ranging from 1.25×0.3NA dry lenses to $25\times$ XL pan N 1.05NA water immersion. A Ti:Sa infra-red (IR) laser line tuned to 920nm is applied to excite the RFP and GFP samples using line switching to minimize crosstalk between fluorochromes. Z-stacks are acquired at 0.5μm increments through the depth of the tumour and vessel area up to 750nm. Images are reconstructed in 3D using Imaris v.5.5.1 (Bitplane, Inc.; St. Paul, Minnesota, USA) or Avizo® v6.2 (Visualization Sciences Group; Burlington, MA, USA).

Parameters

The following parameter values are applied in the simulation.

Table 1. Parameters for the multiscale model, common to the whole simulation, to all proliferating cells, etc. (See [1, 4, 10–12])

Parameter	Default value
Δt	30 min
Δx	40 μm
Domain size	variable
$[\text{Cdh1}]_0$	0.9
$[\text{cycCDK}]_0$	0.01
M_0	5.0
$[\text{p27}]_0$	0.0
$[\text{npRB}]_0$	0.0
b_1	1.0 min^{-1}
b_3	10.0 min^{-1}
J_3	0.04
b_4	35.0 min^{-1}
J_4	0.04
a_4	0.04 min^{-1}
a_2	1.0 min^{-1}
a_3	0.25 min^{-1}
η	0.005 min^{-1}
M^*	10.0
c_2	0.01 min^{-1}

Parameter	Default value
B	0.01
d_2	0.1 min^{-1}
d_1	0.01 min^{-1}
$[\text{VEGF}]_0$	0.0
$[\text{p53}]_0$	0.0
k_7	0.002 min^{-1}
$k_7^{'}$	0.01 min^{-1}
C_{p53}	0.01
k_8	0.002 min^{-1}
J_5	0.04
$k_8^{'}$	0.01 min^{-1}
C_{VEGF}	0.01
P_{O2}	3500
P_{VEGF}	11400
δ_{VEGF}	10.0
D_{O2}	0.00145
D_{VEGF}	0.00145
R_{ex}	5

Table 2. Parameter values that differ for normal and cancer cells, where a dash indicates a parameter that is not defined for that cell type [1, 4, 10–12].

Parameter	Normal cell	Cancer cell
a_1	0.05 min^{-1}	0.4 min^{-1}
c_1	0.1 min^{-1}	0.007 min^{-1}
χ	1.0	0.0
$k_8^{''}$	-0.002 min^{-1}	0.002 min^{-1}
ρ_{THR}	0.75	–
D_m	1	1
Cdh1_{THR}	0.004	0.05
$\text{cycCDK}_{\text{THR}}$	0.2	0.05
$\text{p53}_{\text{THR}}^{\text{high}}$	0.8	–
$\text{p53}_{\text{THR}}^{\text{low}}$	0.08	–
T_{death}	–	4000 min
p27_e	–	1.05
p27_l	–	1.0
V_{THR}	0.27	–
$k_{\text{VEGF}}(\mathbf{x})$	0.3	0.3

Table 3. Vasculature parameters [1, 4, 10–12]

Parameter	Default
P_{in}	35 mmHg
P_{out}	15 mmHg
H_{in}	0.45
μ_0	1.2 g cm min^{-2}
α	0.0
k_s	1.15 s^{-1}
k_p	1.0 s^{-1}
k_m^0	$3.\dot{3}$ s^{-1}
k_m^{VEGF}	0.0
V_0	10^{-3}
τ_{ref}	0
$\dot{Q}_{\text{ref}}$	4×10^{-5} $\text{cm}^3\text{min}^{-1}$
α_R	3.3×10^{-6}
R_{min}	1 μm
R_{max}	50 μm
τ_w^{crit}	$8.\dot{3}$ dynes cm^{-2}
T_{prune}	4000 min
a	0.01

Table 4. Parameters for angiogenic sprouting, or parameters that extend to endothelial cells in addition to normal and cancer cells [1, 4, 10–12].

Parameter	Normal	Cancer	Endothelial Cell
$k_{02}(\mathbf{x})$	13	20	5
D	0	$53.\dot{3}$ μm^2 min^{-1}	$53.\dot{3}$ μm^2 min^{-1}
γ	0	0	2×10^3 μm
N_m	1	1	2
E_m	–	–	2
$P_{\text{sprout}}^{\text{max}}$	–	–	$0.\dot{3} \times 10^{-3}$ min^{-1}
V_{sprout}	–	–	0.001
M_c	–	50	10

Table 5. Parameter values that have been changed for three-dimensional simulations.

Simulation in Figure	Parameter	Value
all	P_{02}	3800
3-8	R_{ex}	3
3-8	T_{prune}	8000 min
11	t_{crit}	30 h
14	γ	2×10^4 μm

References

1. Alarcón T, Byrne HM, Maini PK (2005) A multiple scale model for tumor growth. Multiscale Model Sim 3: 440-475.

2. Tyson JJ, Novak B (2001) Regulation of the eukaryotic cell cycle: Molecular antagonism, hysteresis, and irreversible transitions. J Theor Biol 210: 249-263.

3. Alarcón T, Byrne HM, Maini PK (2004) A mathematical model of the effects of hypoxia on the cell-cycle of normal and cancer cells. J Theor Biol 229: 395–411.

4. Owen MR, Alarcón T, Maini PK, Byrne HM (2009) Angiogenesis and vascular remodelling in normal and cancerous tissues. J Math Biol 58: 689-721.

5. Anderson ARA (2005) A hybrid mathematical model of solid tumour invasion: the importance of cell adhesion. Math Med Biol 22: 163-186.

6. Stokes CL, Lauffenburger DA (1991) Analysis of the roles of microvessel endothelial cell random motility and chemotaxis in angiogenesis. J Theor Biol 152: 377–403.

7. Plank MJ, Sleeman BD (2003) A reinforced random walk model of tumour angiogenesis and anti-angiogenic strategies. Math Med Biol 20: 135–181.

8. Pries A, Reglin B, Secomb T (2001) Structural adaptation of microvascular networks: functional roles of adaptive responses. Am J Physiol 281: H1015-H1025.

9. Pries AR, Secomb TW, Gaehtgens P (1998) Structural adaptation and stability of microvascular networks: theory and simulations. Am J Physiol 275: H349–H360.

10. Alarcón T, Byrne HM, Maini PK (2003) A cellular automaton model for tumour growth in an inhomogeneous environment. J Theor Biol 225: 257-274.

11. Alarcón T, Owen MR, Byrne HM, Maini PK (2006) Multiscale modelling of tumour growth and therapy: the influence of vessel normalisation on chemotherapy. Comput Math Method M 7: 85-119.

12. Betteridge R, Owen MR, Byrne HM, Alarcón T, Maini PK (2006) The impact of cell crowding and active cell movement in vascular tumour growth. Networks and Heterogenous Media 1: 515-535.
